# Association between the Dynamics of Multiple Replication Origins and the Evolution of Multireplicon Genome Architecture in Haloarchaea

**DOI:** 10.1093/gbe/evu219

**Published:** 2014-10-03

**Authors:** Zhenfang Wu, Haibo Yang, Jingfang Liu, Lei Wang, Hua Xiang

**Affiliations:** ^1^State Key Laboratory of Microbial Resources, Institute of Microbiology, Chinese Academy of Sciences, Beijing, China; ^2^University of Chinese Academy of Sciences, Beijing, China

**Keywords:** multiple replication origins, origin dynamics, evolution, genome architecture, halophilic archaea

## Abstract

Haloarchaeal genomes are generally composed of multiple replicons, and each replicon has a single or multiple replication origin(s). The comparative genomic analysis of replication origins from closely related species can be used to reveal the evolutionary mechanisms that account for the development of multiple origin systems. Multiple replication origins have been in silico and experimentally investigated in *Haloarcula hispanica*, which raise the possibility for comparisons of multiple replication origins in *Haloarcula* species. Thus, we performed a comparison of *H. hispanica* replication origins with those from five additional *Haloarcula* species. We demonstrated that the multiple replication origins in the chromosome were evolved independently multiple times from the *oriC1*-dependent ancestral chromosome. Particularly, the two origins *oriC1* and *oriC2* were conserved in location, and both of them were adjacent to an rRNA operon, suggestive of correlations in replication and expression of surrounding genes that may promote the conservation of these two origins. Some chromosomal variable regions were used as hotspots for origin evolution in which replication origins were continually being acquired, lost, and disrupted. Furthermore, we demonstrated that autonomously replicating sequence plasmids with *H. hispanica* minichromosomal replication origins were extremely unstable. Because both organization and replication origins of minichromosomes were not conserved, we proposed an association between the evolution of extrachromosomal replicons and origin variation. Taken together, we provided insights into the evolutionary history of multiple replication origins in *Haloarcula* species, and proposed a general model of association between the dynamics of multiple replication origins and the evolution of multireplicon genome architecture in haloarchaea.

## Introduction

Similar to bacteria, archaea contain circular chromosomes and initiate chromosome replication at specific sites known as replication origins (Robinson and Bell 2005). However, despite the first description of archaeal replication origins demonstrated that the chromosome of the hyperthermophilic archaeon *Pyrococcus abyssi* uses a single origin to initiate replication (Myllykallio et al. 2000; Matsunaga et al. 2001, 2003), many archaea characterized to date harbor multiple discrete replication origins (Lundgren et al. 2004; Norais et al. 2007; Robinson and Bell 2007; Coker et al. 2009; Pelve et al. 2012, 2013; Wu et al. 2012, 2014; Hawkins et al. 2013). Among archaea, multiple replication origins have been described in great detail in *Sulfolobus* species, providing insights into the characterization, utilization, and evolution of the three active replication origins in their single chromosome (Robinson et al. 2004; Dueber et al. 2007; Robinson and Bell 2007; Duggin et al. 2008; Samson et al. 2013). The characterized archaeal origins are normally conserved in structure but vary in sequence among different origins in terms of origin recognition boxes (ORBs) and origin-associated initiator genes (Wu et al. 2012). Recently, the specific recognition of initiator genes to their cognate origins was experimentally established in *Sulfolobus islandicus* (Samson et al. 2013) and *Haloarcula hispanica* (Wu et al. 2014). The origins together with their adjacent initiator genes are considered to be distinct replicator-initiator systems, and the integration of extrachromosomal elements has been proposed to account for mosaics of multiple replication origins in specific archaeal chromosomes (Robinson and Bell 2007; Wu et al. 2012). This inferred that the specific linkage between the ORB elements and the corresponding initiator gene is conserved during a long-term evolution, and such a conserved replicator-initiator pairing may translocate frequently among different species.

Haloarchaea are a distinct group of archaea that thrive in hypersaline environments. Haloarchaeal genomes are generally distributed among several replicons, and each replicon has a single or multiple replication origin(s) (Capes et al. 2011), which complicates our understanding of their replication characteristics and evolutionary history. Recently, we performed an in silico study to predict replication origins, and the results demonstrated that the occurrence of multiple replication origins is widespread in haloarchaea and that up to seven putative origins are located on the *Haloterrigena turkmenica* chromosome (Wu et al. 2012). Furthermore, the active origins have been experimentally studied in three model systems: *Halobacterium* sp. strain NRC-1 (Berquist and Dassarma 2003; Coker et al. 2009), *Haloferax volcanii* (Norais et al. 2007; Hawkins et al. 2013), and *H. hispanica* (Wu et al. 2012, 2014). Remarkably, replication origins are highly diverse in both sequence and utilization in haloarchaea. Unexpectedly, the number of predicted origins was normally greater than that of active origins in each characterized strain, particularly in the extrachromosomal replicons. Thus, it is intriguing to investigate the evolutionary processes that accounted for the development of multiple replication origins in haloarchaea. Insertion, deletion, and genome rearrangement occurred frequently in haloarchaea (Dyall-Smith et al. 2011), and we demonstrated that replication origins were transferred frequently among different haloarchaea (Wu et al. 2012). In addition, a comparative genomic analysis of the replication origins in the chromosomes of *H. hispanica* and *Haloarcula marismortui* revealed that strain-specific origins are located in the chromosomal divergent regions (Wu et al. 2012). Thus, there might be correlations between origin diversity and genome variation. Comparative genomic analyses of replication origins have been performed to address the evolution of the replication origins at the structural, locational, and regulatory levels in budding yeasts (Di Rienzi et al. 2012; Muller and Nieduszynski 2012). Thus, a comparison of the replication origins from closely related haloarchaeal species should reveal the evolutionary processes responsible for the development of multiple origins in haloarchaea.

We have previously investigated the utilization of multiple replication origins in *H. hispanica*. Although both the main chromosome and minichromosome use two active replication origins in vivo, one active replication origin per replicon is sufficient for genome replication (Wu et al. 2012, 2014). The two active replication origins in the chromosome were proposed to originate from integration of the *oriC2-cdc6E* into an ancestral chromosome that was dependent on *oriC1-cdc6A* (Wu et al. 2014). In addition, three replication origins, *oriC3-cdc6D* in the chromosome and *oriC4-cdc6G* and *oriC5-cdc6H* in the minichromosome, were proven to be nonfunctional and were considered to be deficient or dormant replication origins (Wu et al. 2012, 2014). To further address how multiple replication origins evolved over evolutionary time, in this study, we performed a comparative genomic analysis of the *H. hispanica* replication origins with those obtained from five additional *Haloarcula* species, specifically focusing on the dissection of the three *H. hispanica* nonfunctional origins. These comparative analyses demonstrated frequent variation of the replication origins in both chromosomal variable regions and unstable extrachromosomal replicons, while the conserved replication origins were maintained in location which may be promoted by their impacts on surrounding genes or on the stabilization of minireplicons. Comparative analyses of the three *H. hispanica* deficient origins with their homologs in other strains demonstrated that all of them were deficient, which might be due to structural destruction accompanying the frequent insertion and deletion events in the chromosomal variable regions or the frequent rearrangement of variable minireplicons.

## Materials and Methods

### Strains, Plasmids, and Culture Conditions

*Escherichia coli* cells were grown in Luria–Bertani medium at 37 °C, and 100 μg/ml ampicillin was added when required. *H. hispanica* and *H. marismortui* strains were cultured at 37 °C in nutrient-rich medium AS-168 as previously described (Wu et al. 2012). When required, 3 μg/ml mevinolin was added. The investigation of the autonomous replication ability of replication origins was based on the plasmid pBI101 (Zhou et al. 2007). pBI101 plasmids containing the replication origins pOC1, pOC2, pOC6, pOC7, and pOP were previously constructed (Wu et al. 2014).

### Autonomous Replication Ability Assay

The plasmid-based assay of autonomously replicating sequence (ARS) activity was performed as previously described (Wu et al. 2012). In each assay, the origin region together with the *cdc6* coding region was amplified from *H. hispanica* or *H. marismortui* genomic DNA and was cloned into the nonreplicating plasmid pBI101. After sequencing, the plasmids were then introduced into *H. hispanica* or the corresponding origin-deletion strains (Wu et al. 2014) using a polyethylene glycol-mediated transformation method (Cline et al. 1989), and the mevinolin-resistant transformants were selected on AS-168 plates with 3 μg/ml mevinolin.

### Estimation of ARS Plasmid Stability

The estimated stability of the ARS plasmid with different replication origins was performed as previously described (Norais et al. 2007). The five ARS plasmids were transformed into *H. hispanica* or the corresponding origin-deletion strains using a polyethylene glycol-mediated transformation method (Cline et al. 1989), and the mevinolin-resistant transformants were selected on AS-168 plates with 3 μg/ml mevinolin. For each transformation, a colony containing the ARS plasmid was selected and inoculated into 10 ml of AS-168 (Mev) broth under selection. The cultures were then propagated twice in AS-168 broth without mevinolin, and at each passage, the cultures were plated on AS168 plates. Finally, the colonies were patched on an AS168 (Mev) plates to determine the fraction of mevinolin-resistant cells.

### Genome Resources and Comparative Genomics

Three completed genome sequences, namely those of *H. hispanica* ATCC 33960 (Liu et al. 2011), *H. hispanica* N61 (Ding et al. 2014), and *H. marismortui* ATCC 43049 (Baliga et al. 2004), and contigs from four draft genomes (Lynch et al. 2012), namely those of *Haloarcula amylolytica* JCM 13557, *Haloarcula argentinensis* DSM 12282, *Haloarcula japonica* DSM 6131, and *Haloarcula sinaiiensis* ATCC 33800, were available through The National Center for Biotechnology Information (NCBI) (http://www.ncbi.nlm.nih.gov/genome/). Genome visualization and comparative genomics were performed using the CGView Server (Grant and Stothard 2008) with default parameters (http://stothard.afns.ualberta.ca/cgview_server/).

### Distribution and Contextual Analysis of Replication Origins

The distribution of the *H. hispanica* replication origins in *Haloarcula* species was performed via BLASTP analysis (BLOSUM62 matrix; 1 × 10^−6^ as an *e* value cutoff) of origin-associated Cdc6 proteins against a specific *Haloarcula* genome (http://blast.ncbi.nlm.nih.gov/) (Altschul et al. 1990). Genome context analysis was performed using the NCBI Genome Workbench and scrutinized manually. Pairwise and multiple alignments of replication origins or Cdc6 proteins were generated using the DNAMAM software (for Windows, version 2.6). The identification of ORB elements in the replication origins was performed via motif searches using the MEME software (motif size: 20-40; ZOOPS model) (Bailey et al. 2006).

### Phylogenetic Analysis

The RNA polymerase B' subunit (RpoB') was used for phylogenetic analysis as described (Minegishi et al. 2010). The RpoB' amino acid sequences were collected, and multiple alignments were generated using Clustal (substitution matrix = BLOSUM; gap-opening penalty = 10; gap-extension penalty = 0.1). Maximum-likelihood phylogeny was performed with PHYML v3.0 with an LG substitution model and 100 nonparametric bootstrap replicates (Guindon et al. 2010).

## Results

### Dynamics of Multiple Replication Origins in *Haloarcula* Species

Recently, multiple replication origins have been in silico predicted and experimentally confirmed in *H. hispanica* (Wu et al. 2012, 2014). To further understand the evolutionary history of these multiple replication origins, a comparison of replication origins of *H. hispanica* was performed with those of five additional *Haloarcula* species that are closely related to *H. hispanica*. Thus, we first analyzed the distribution of the *H. hispanica* replication origins in *Haloarcula* species using BLASTP analyses of the eight Cdc6 proteins associated with putative replication origins against the following five *Haloarcula* species: *H. amylolytica*, *H. argentinensis*, *H. japonica*, *H. marismortui*, and *H. sinaiiensis* (table 1)*.* There were three conserved replication origins in *Haloarcula* species: *oriC1-cdc6A*, *oriC2-cdc6E*, and *oriP-cdc6K* (table 1). Previous studies (Robinson et al. 2004; Coker et al. 2009; Wu et al. 2012; Raymann et al. 2014) have demonstrated that the *oriC1-cdc6A* is broadly conserved across the archaeal domain of life, which has been considered to be inherited from the ancestor of archaea. In contrast, the *oriC2-cdc6E* and *oriP-cdc6K* were not present in all haloarchaea; thus, the conservation of these two replication origins appeared to be restricted to *Haloarcula* species, indicating that the acquisition of these two replication origins by *Haloarcula* occurred before the divergence of individual strains. The remaining five replication origins were either present or absent in an individual strain, that is, the distribution of these origins was variable in *Haloarcula* species (table 1). It is possible that these variable replication origins can be attributed to strain-specific origin gains and losses, which would account for the diversity of replication origins in *Haloarcula* species.

**Table 1:** Distribution of *H. hispanica* Putative Replication Origins in Five Additional *Haloarcula* Species

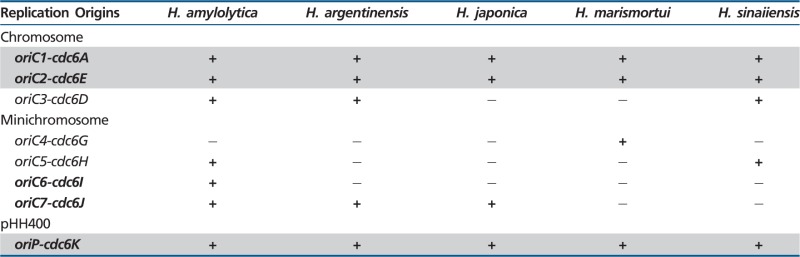

Note.—The plus (+) and minus (−) signs indicate the presence and absence, respectively, of the *H. hispanica* replication origins in the other five *Haloarcula* genomes. The five functional replication origins of the eight putative origins in *H. hispanica* are indicated in bold. The conserved replication origins in *Haloarcula* species are highlighted with a gray background.

Taken together, these findings show that multiple replication origins are dynamic in *Haloarcula* species, and these origins could be separated into two categories: conserved and variable. The conserved replication origins were present in all of the analyzed strains, and are thus likely inherited from the original archaeal ancestor or a *Haloarcula* ancestor and maintained stable. The variable origins were not found in all of the strains, and this may be due to strain-specific acquisition or deletion events.

### Maintenance of the Conserved Replication Origins via Their Impacts on Surrounding Environments

**Fig. 1.—:**
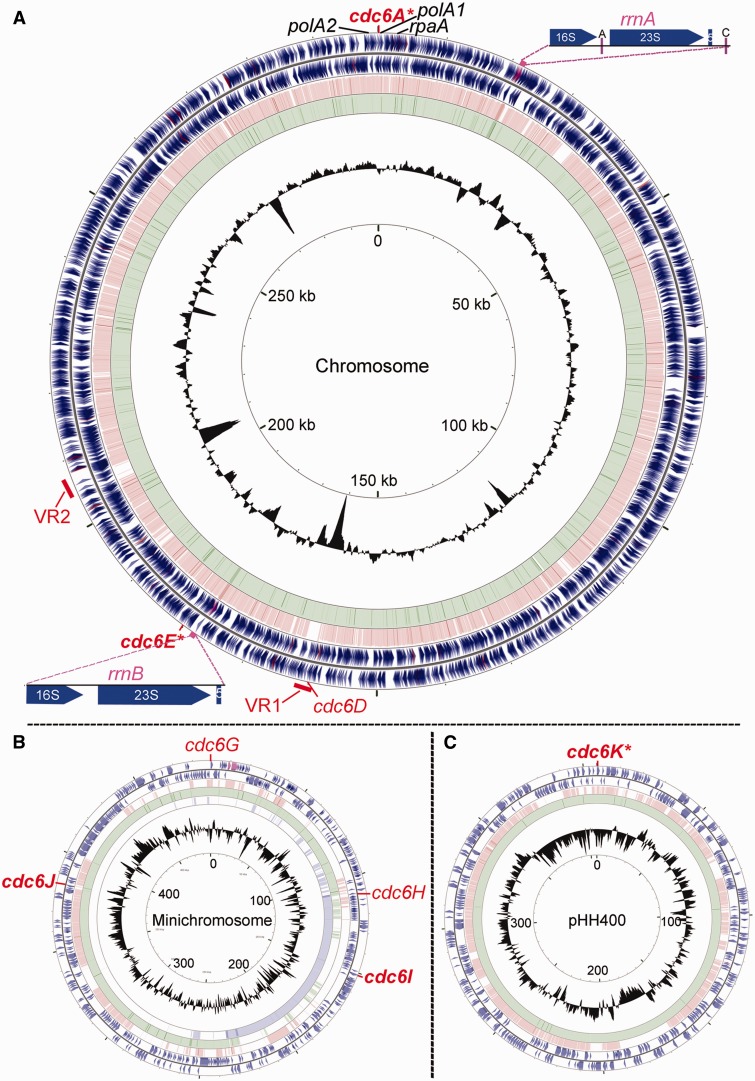
Comparative genomic analysis of the replication origins in *Haloarcula* species. (*A*) The chromosome. From the outside inward: The first and second circles indicate the annotated protein-coding regions on the plus and minus strands in *H. hispanica* ATCC 33960. The third and fourth circles represent the BLASTN hits of the chromosome of *H. hispanica* ATCC 33960 with those of two other *Haloarcula* genomes: *H. marismortui* ATCC 43049 and *H. hispanica* N61, respectively. The innermost circle shows a plot of the GC content along the chromosome of *H. hispanica* ATCC 33960. The two large variable regions (VR1-2, red) and the positions of two *rrn* operons are indicated. The two *rrn* operons are both directed away from the origins. (*B* and *C*) Minichromosome and megaplasmid pHH400. The circles in *B* and *C* are the same as in *A* with the exception that the *H. hispanica* N61 minichromosome was divided into two replicons: The minichromosome and pHH126 (the fourth and fifth circles in *B*). In all three replicons, the *cdc6* genes associated with the putative replication origins in *H. hispanica* are indicated, and those with active origins are indicated in bold. The conserved *ori-cdc6* origins in *Haloarcula* species, namely *oriC1-cdc6A* and *oriC2-cdc6E* on the chromosome and *oriP-cdc6K* on PHH400, are highlighted with star signs. GC, Guanine-Cytosine.

The two conserved replication origins *oriC1-cdc6A* and *oriC2-cdc6E* were located in the main chromosome of *Haloarcula* species (fig. 1*A*). Our previous marker frequency analysis (MFA) results demonstrated that both of these two replication origins are active in vivo and that each of these replication origins is sufficient for chromosome replication (Wu et al. 2014). Thus, it raises the question of why these origins were conserved over evolutionary time, particularly when we proposed that *oriC2-cdc6E* appeared to be acquired in the branch leading to *Haloarcula*. To address this question, we examined the common characteristics between the conserved replication origins, which might play important roles on their evolutionary conservation. We found that *oriC1-cdc6A* and *oriC2-cdc6E* are conserved in their chromosomal location (fig. 1*A*), suggesting that these two conserved origins might be promoted via their surrounding environments. This hypothesis is reinforced by the cluster of replication-associated genes, such as DNA polymerase genes (*polA1* and *polA2*) and replication protein A (*rpaA*) (fig. 1*A*), around the *oriC1-cdc6A* origin. In addition, we found that an rRNA operon (*rrn* operon) was positioned downstream of each origin, and these two *rrn* operons are approximately 211 and 20 kb away from *oriC1-cdc6A* and *oriC2-cdc6E*, respectively (fig. 1*A*). Similar genomic organization that *rrn* operons are close to (and transcribed away from) the two chromosomal replication origins has also been observed in *Haloferax volcanii* (Norais et al. 2007; Hartman et al. 2010). This organization may ensure that the two *rrn* operons (and other genes closer to the origins) are able to be replicated earlier compared with the rest of the genome, thereby enhancing the expression of these genes due to a gene dosage effect. In addition, the concurrent rounds of DNA replication has been indicated in *Haloferax volcanii* (Hawkins et al. 2013), which would be expected to further amplify the dosage. Conversely, high expression of replication-associated genes (such as *cdc6* genes) contributes to active utilization of origins that may promote their conservation. Furthermore, both the two *rrn* operons are directed away from the origins (fig. 1*A*), which may ensure codirection of replication and transcription that improves the stability of surrounding genes (Paul et al. 2013). We proposed that, similar to *oriC1-cdc6A*, the effects of the *oriC2-cdc6E* on surrounding genes (gene expression or gene stability) after its acquisition in the *Haloarcula* ancestor may promote its conservation in the divergence of individual strains. The other conserved replication origin was *oriP-cdc6K* from the megaplasmid pHH400, and this replicon was considered to be stable (fig. 1*C*), suggesting that the conservation of *oriP-cdc6K* might ensure the stability of pHH400 in *Haloarcula* species. Based on these findings, we proposed that origin conservation may be promoted by the effects of these origins on their surrounding environments, which may explain why conserved replication origins are also active in vivo (table 1).

### Replication Origins Evolved Frequently in Genomic Variable Regions

In contrast with conserved replication origins, variable replication origins are normally strain-specific and are located in divergent regions of the genome. We previously proposed that these origins were recently acquired via translocation events (Wu et al. 2012). Thus, we considered that the characterization of these variable origins might contribute to our understanding of the evolutionary history of the development of multiple replication origins in haloarchaeal genomes and of the association between origin diversity and genome variation. To address this hypothesis, analyses of the localization and genome context of the variable origins were performed.

**Fig. 2.—:**
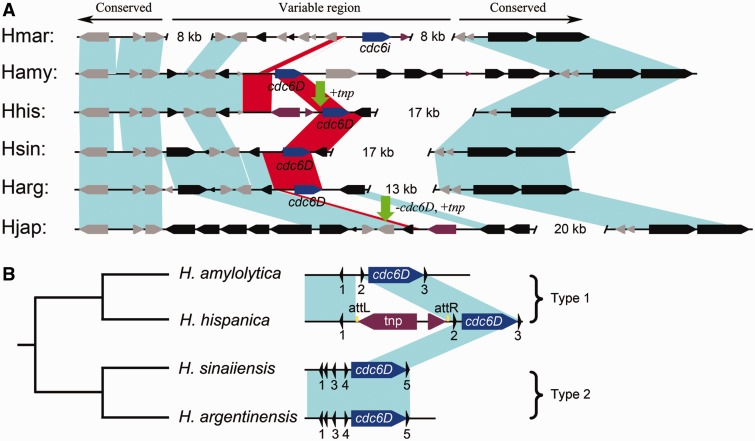
Replication origins evolved frequently in chromosomal variable regions. (*A*) Sequence similarity and gene order analyses of the chromosomal variable region VR1. The *cdc6* homologs in VR1 are indicated in blue. The variable regions and the edges of the conserved regions were confirmed using BLASTN alignments of the sequences, and a sequence similarity greater than 70% is represented with teal shading; the red-shaded regions indicate homologous regions around the *cdc6D* gene. The vertical green arrows indicate that the local regions were disrupted by putative transposases (shown in purple). (*B*) Two types of *cdc6D*-associated replication origins in VR1. (Left) Phylogenetic relationship of the four *Haloarcula* species (not to scale). (Right) Physical map of the *cdc6D*-associated replication origins; the ORB motifs are indicated with small triangles. The shaded teal regions denote a similarity greater than 70%, as determined through BLASTN analyses, and the attachment (att) sites for the insertion of a putative transposase within the *oriC3-cdc6* origin of *H. hispanica* are indicated in yellow.

The third replication origin (*oriC3-cdc6D*) in the *H. hispanica* chromosome was not conserved in *Haloarcula* species because its homologs were only observed in three other species, *H. amylolytica*, *H. argentinensis*, and *H. sinaiiensis* (table 1). The chromosomes of *H. hispanica* and *H. marismortui* were completely collinear with only two large regions of species-specific variation, and the *cdc6D* gene was located in VR1 (Wu et al. 2012 and fig. 1*A*). A detailed comparison with four additional *Haloarcula* species revealed that this region is highly variable and is located in exactly the same position relative to the conserved regions in each chromosome (fig. 2*A*). This observation suggests that this region is a hotspot for the integration of foreign sequences, that is, it is a location in which insertion and deletion events occurred frequently, and this conclusion was supported by the distribution of transposases in this region of each chromosome (fig. 2*A*). Interestingly, similar to the situation in *H. hispanica*, the *cdc6D* homolog was also located in this variable region in *H. amylolytica*, *H. argentinensis*, and *H. sinaiiensis* (fig. 2*A*). It was suggested that the independent gain and loss of *oriC3-cdc6D* homologs in this frequently variable region (VR1) might account for the variation of this origin among *Haloarcula* chromosomes.

All four replication origins located in the *H. hispanica* minichromosome, including the two functional origins *oriC6-cdc6I* and *oriC7-cdc6J*, were not conserved in *Haloarcula* (table 1). The minichromosome appeared to be highly dynamic in *Haloarcula* species, as well as the two laboratory-derived *H. hispanica* genomes, *H. hispanica* ATCC 33960 and *H. hispanica* N61 (fig. 1*B*). The 489-kb minichromosome in *H. hispanica* ATCC 33960 appeared to be divided into two replicons in *H. hispanica* N61, a 363-kb minichromosome and a 126-kb pHH126. Nevertheless, large and small pairs of orthologs were observed throughout the comparison (fig. 1*B*). These observations indicate that this replicon was not stable and was reconstructed frequently in the divergence of the species. Thus, we proposed that the frequent reconstruction of the minichromosome might account for the variable replication origins in this replicon because independent gains and losses of replication origins accompanied the reconstruction processes. In addition, *oriC4-cdc6G* and *oriC5-cdc6H* were confirmed to be nonfunctional, which might be due to their incomplete acquisition or functional disruption during the reconstruction of the minichromosome.

Taken together, these findings revealed a rapid evolution of replication origins in the variable genome regions during the integration of foreign sequences at chromosomal variable regions or the reconstruction of extrachromosomal replicons, suggesting that variable replication origins might be associated with genomic variable regions.

### Addition, Deletion, and Disruption of Replication Origins in Chromosomal Variable Regions

As previously described, the replication origins in the variable regions evolved frequently; thus, these variable origins could serve as models for evolutionary mechanisms responsible for origin diversity. We addressed the evolutionary characteristics of *cdc6D*-associated replication origins by analyzing the origin region directly upstream of the *cdc6D* gene in each genome. Interestingly, although the ORB elements showed high sequence conservation, two types of origins with different structures were observed. In particular, the *cdc6D*-associated origins showed high conservation between *H. hispanica* and *H. amylolytica* (type 1), or between *H. sinaiiensis* and *H. argentinensis* (type 2); however, these two types of *cdc6D*-associated origins showed limited conservation both in sequence and in structure (fig. 2*B*). These results indicated that, although the linkage-specificity of the ORB sequences and Cdc6D protein was conserved over long evolutionary distances, the structure of the origin was only conserved over short evolutionary distances. More importantly, in conjunction with the gene order analysis, we proposed that the *cdc6D*-associated origin was integrated at this variable region in different chromosomes from two independent origin gains. Integration of extrachromosomal elements that accounts for replicon evolution has been previously proposed in *Aeropyrum pernix* (Robinson and Bell 2007). The *oriC3-cdc6D* of *H. hispanica* was disrupted by a putative transposase in *H. hispanica* compared with that of *H. amylolytica*. In addition, the *cdc6D*-associated origin of *H. japonica* appeared to be replaced by a putative transposase via coupled insertion and deletion events compared with that of *H. argentinensis*. Thus, we concluded that frequent strain-specific addition, deletion, and disruption events in the variable region accounted for the diversity of the *oriC3-cdc6D* origin in different chromosomes. A distinct origin associated with *cdc6i* was observed in this variable region in the *H. marismortui* chromosome, which supports the frequent acquisition of replication origins in variable regions. It is also likely the explanation that a *cdc6g*-associated origin was observed in the other variable region (VR2) of the chromosome in *H. marismortui* but not in *H. hispanica* (Wu et al. 2012). Taken together, the results suggest that replication origins evolved multiple times in the variable regions of the chromosome and were continually being created and destroyed.

### ARS Plasmids with Minichromosomal Replication Origins Are Significantly Unstable

**Fig. 3.—:**
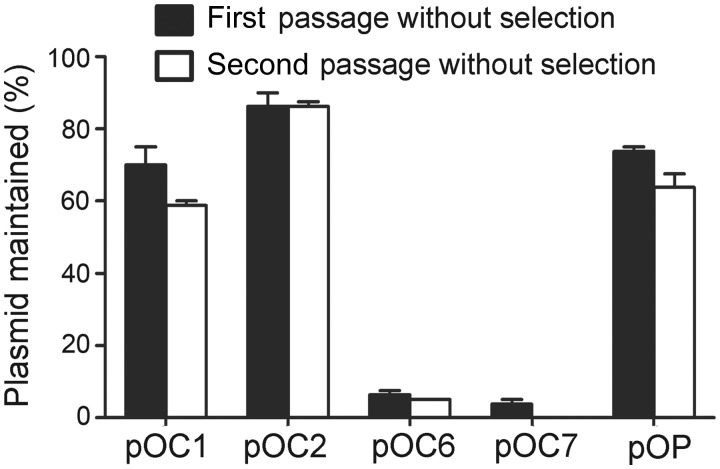
Instability of ARS plasmids with the two minichromosomal origins *oriC6* and *oriC7*. Transformants with pOC1, pOC2, pOC6, pOC7, and pOP were propagated in nonselective AS168 broth for two passages. At each passage, the cultures were plated on AS168 medium, and the colonies were patched on AS168 (Mev) selective plates to determine the fraction of mevinolin-resistant cells.

In *Haloarcula* species, we demonstrated that both the organization and replication origins of the minichromosome are variable, but pHH400 and its origin are stable. Thus, we proposed that there might be correlations between the replication origins and the fate of extrachromosomal replicons. To address this question, we determined the stabilities of ARS plasmids with the five functional replication origins of *H. hispanica*: *oriC1* (pOC1) and *oriC2* (pOC2) from the chromosome, *oriC6* (pOC6) and *oriC7* (pOC7) from the minichromosome, and *oriP* (pOP) from pHH400. Transformants containing each of these five ARS plasmids were propagated in AS168 broth without mevinolin selection and passaged every 4 days. At each passage, the cultures were diluted and plated on AS168 nonselective medium, and the colonies were subsequently patched on mevinolin-selective plates to determine the fraction of mevinolin-resistant cells. As shown in figure 3, compared with pOC1, pOC2, and pOP, the pOC6 and pOC7 were significantly less stable. After two passages, the *oriC6* plasmid pOC6 was maintained in only 5% of the colonies, and the *oriC7* plasmid pOC7 was completely lost (fig. 3). Importantly, our results demonstrated that the *oriC6*- and *oriC7*-containing ARS plasmids were significantly less stable compared with the *oriP*-containing plasmid, which suggests that the minichromosomal replication origins are less able to maintain replicon stability than the origin from pHH400. Thus, we proposed that the instability of *oriC6*- and *oriC7*-containing ARS plasmids might reflect the unstable nature of the minichromosomes in *Haloarcula* species, which conversely forced the variation of their bearing replication origins, suggesting an association between the evolution of extrachromosomal replicons and the bearing replication origins.

### Risk Factors for Variable Replication Origins

**Fig. 4.—:**
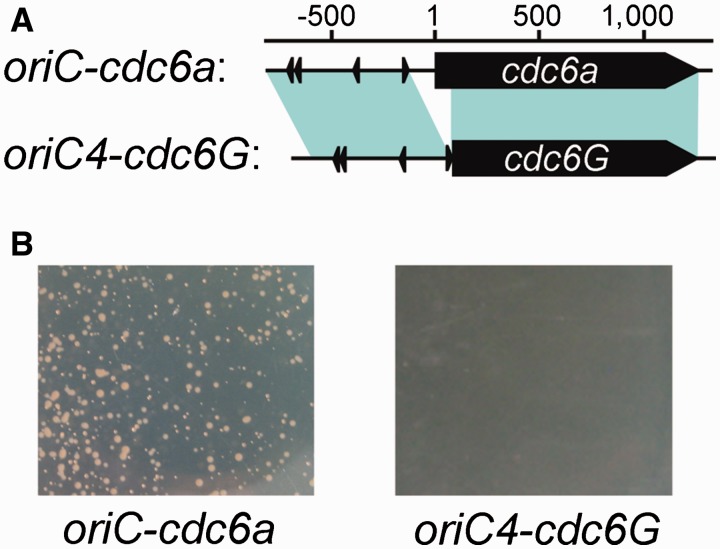
Loss of *cdc6* promoter broke the ARS activity of the *H. hispanica oriC4-cdc6G*. (*A*) Sequence alignment and physical mapping of *H. hispanica oriC4-cdc6G* and its *H. marismortui* homolog *oriC-cdc6a*. The *cdc6* genes are indicated, and the start site of *H. marismortui cdc6a* is numbered one. The ORB elements are indicated with arrowheads. The shaded teal regions denote a similarity greater than 70%, as determined through BLASTN analyses. These two replication origins were highly conserved with the exception of a 203-bp sequence between the *cdc6* gene and the origin region, which was lost in *H. hispanica oriC4-cdc6G*. (B) ARS assay plates for the two origins. Colonies in plates of AS168 (Mev) were observed after 7 days at 37 °C.

**Fig. 5.—:**
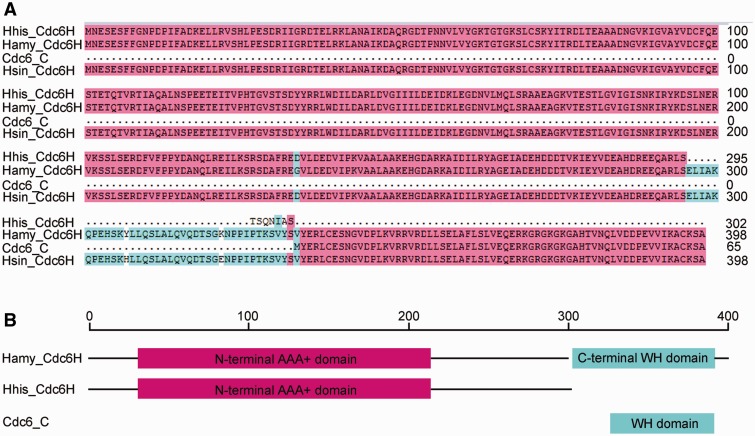
Disruption of the integrity of the Cdc6H protein in *H. hispanica*. (*A*) Multiple alignments of *H. hispanica* (Hhis), *H. amylolytica* (Hamy), and *H. sinaiiensis* (Hsin) Cdc6H proteins and a small *H. hispanica* Cdc6_C protein. (*B*) Functional domain annotation on Cdc6H homologs generated using the CD-search tool in the NCBI website.

We have revealed the association between origin diversity and genome variation and have confirmed that some of these variable origins are nonfunctional. These findings raise the questions of whether and why frequent evolution of the variable regions affects the function of bearing replication origins. To address this question, we performed comparative analysis to characterize the three *H. hispanica* nonfunctional replication origins with their homologs from other *Haloarcula* species. As previously described, we found that the origin region of *oriC3-cdc6D* was disrupted by an insertion of a putative transposase in *H. hispanica* (fig. 2*B*), which might disrupt the function of this origin. In addition, a homolog of *oriC4-cdc6G*, designated as *oriC-cdc6a*, was found in the minichromosome of *H. marismortui*. The alignment analysis of *oriC4-cdc6G* and *oriC-cdc6a* revealed high homology extended over the full length of the *ori-cdc6* sequence with the exception of a 203-bp sequence loss in *oriC4-cdc6G*. This sequence included the 5′-terminal 75-bp coding region and the entire promoter of the *cdc6* gene (fig. 4*A*). Indeed, the *oriC-cdc6a* origin presented ARS activity because it was able to confer replication ability to a nonreplicating plasmid (fig. 4*B*). Thus, *oriC-cdc6a* might be the active origin for replication of the *H. marismortui* minichromosome, which explains why neither of the two functional origins of the *H. hispanica* minichromosome was observed in the *H. marismortui* minichromosome (table 1). The comparison analysis of *oriC5-cdc6H* from different species revealed the lack of the C-terminal winged-helix (WH) domain of Cdc6H in *H. hispanica* (fig. 5). Thus, the *oriC4-cdc6G* and *oriC5-cdc6H* origins in *H. hispanica* did not contain an intact functional initiator gene, and this absence is a highly likely explanation of why they do not exhibit origin activity. Furthermore, we found a perfect hit to the C-terminal WH domain of Cdc6H in the *H. hispanica* minichromosome, a small Cdc6 homolog (HAH_4250, designated Cdc6H_C for simplicity) (fig. 5). Remarkably, there was an approximate 123-kb distance between *cdc6_c* and *oriC5-cdc6H*. Indicators of translocation processes (integrases or transposases) were observed around both of *cdc6_c* and *oriC5-cdc6H*, suggesting that the intact *oriC5-cdc6H* origin was separated and thus disrupted during the construction of the minichromosome in *H. hispanica*. Taken together, as these three *H. hispanica* nonfunctional origins are not conserved and located in variable regions in *Haloarcula* species, we suggested that their integrity was destroyed during genome variation, either due to incomplete acquisition or functional disruption. In addition, our comparison analyses provided us with the ability to understand the essential elements for a functional replication origin, which would greatly contribute to the dissection of mosaic replication origin systems in haloarchaeal genomes.

## Discussion

Multiple replication origins have been previously predicted in most haloarchaeal genomes (Wu et al. 2012), and active origins have been experimentally identified in several model systems (Norais et al. 2007; Coker et al. 2009; Wu et al. 2014). These data indicate the major diversity of replication origins in different strains. Although translocation events have been proposed to account for the mosaics of replication origins in haloarchaeal genomes, the detailed mechanisms of these evolutionary processes are less understood. Recently, multiple replication origins have been in silico and experimentally investigated in *H. hispanica* (Wu et al. 2014). In this manuscript, based on the replication origins in *H. hispanica*, we report the first comparative analysis of replication origins from multiple *Haloarcula* species to understand the evolutionary mechanisms involved in the development of multiple origin systems. Our comparison analyses demonstrated that the dynamics of multiple replication origins is associated with the evolution of multireplicon genome architecture, which indicates that genome evolution forces origin dynamics in variable regions and that maintenance of the conservative replication origins may be promoted by their effects on surrounding genomic environments. This is the first report revealing the dynamics and evolutionary history of multiple replication origins in haloarchaea using comparative genomic analysis of replication origins from closely related species, which not only contributes to understanding of multiple-origin systems in the domain of Archaea but also provides insight into the mechanisms of the more complex replication origins found in eukaryotes.

The comparative analyses revealed that replication origins can be classified into conserved and variable origins in *Haloarcula* species (table 1, fig. 1 and Wu et al. 2012). The conserved origins are present in all species, and our results demonstrated that these origins were conserved not only in sequence and structure but also in their genomic location. However, the variable origins are either present or absent in a single species and are located in the genomic variable regions. These results suggest that the dynamics of multiple replication origins might be associated with genomic contexts. In particular, genome evolution forced frequent variation of replication origins in haloarchaea with the exception of cases in which the origin was conserved via its effects on surrounding genes.

Because origin variation normally occurs in genomic variable regions, it was unclear to what degree these variable replication origins survive in the face of genomic change and whether origin evolution affects genome replication and genome architecture. The comparative dissection of the *H. hispanica* nonfunctional replication origins with their homologs in other species revealed that frequent coupled insertion and deletion events were continually creating and destroying replication origins in genomic variable regions. The rapid evolution of replication origins in variable regions might largely account for origin diversity, and our insights into these variable replication origins thus provide us with the ability to understand the evolutionary mechanisms responsible for mosaic origin systems in haloarchaea. In addition, frequent acquisitions and losses of replication origins might fundamentally alter the manner of genome replication, such as the development of a multiple-origin replicon from a single-origin replicon. Interestingly, an *Haloferax volcanii* strain lacking all active replication origins was constructed, and the recombination-dependent manner of chromosome replication was proposed to be used (Hawkins et al. 2013; Michel and Bernander 2014). The alteration of the manner of replication is very common in reconstructing processes of extrachromosomal replicons, which explains why none of the conserved active origins was observed in the minichromosomes of *Haloarcula* species (fig. 1*B*).

**Fig. 6.—:**
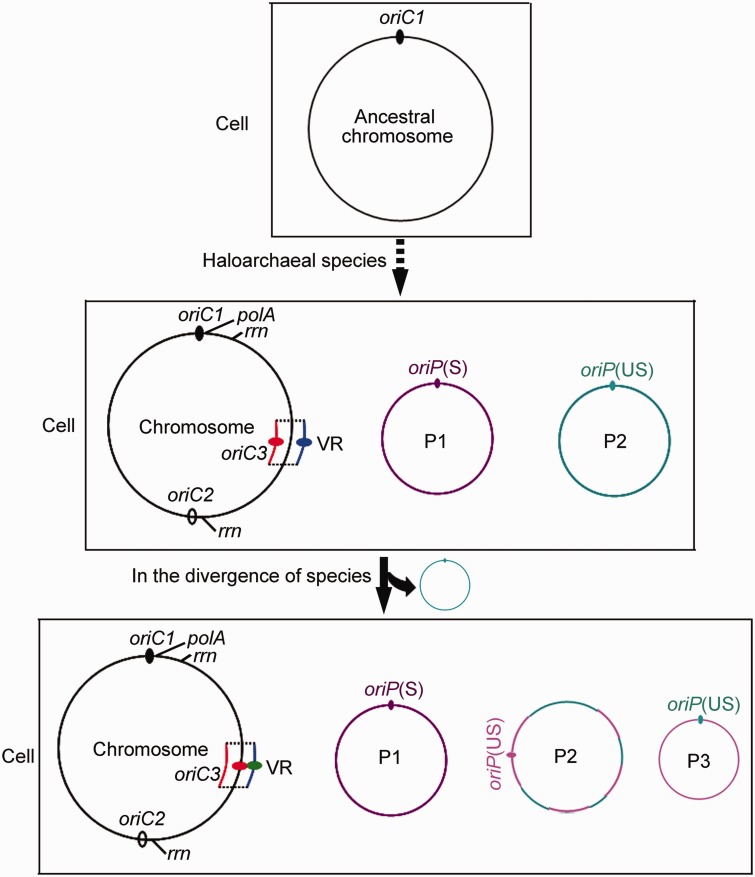
Model of the association between the dynamics of replication origins and genome evolution. The haloarchaeal multireplicon genome evolved in multiple steps (indicated with the dotted arrow) from the *oriC1*-dependent archaeal ancestral chromosome (up). For example, novel replication origins (*oriC2* and *oriC3*) were independently integrated into the chromosome, and minireplicons were constructed in the branch leading to specific haloarchaeal species, whereas *oriC1* was conserved via its effects on surrounding genes (middle). Both the replication origins and the genome architecture (particularly the extrachromosomal replicons) varied frequently in the divergence of species (down). The *oriC2* origin was conserved in a similar manner as *oriC1* via its effects on surrounding genes, whereas *oriC3* was continually being acquired, lost and disrupted in the chromosomal variable regions (in different colors). The fate of extrachromosomal replicons was determined by the hierarchy in the plasmid stabilization of their bearing replication origins. Conversely, unstable extrachromosomal replicons forced variation of their bearing replication origins. For example, the plasmid P1 (in purple) was conserved, as was the stability of the ARS plasmid with its bearing origin *oriP*(S). In contrast, the plasmid P2 (in dark green) was easily lost because the *oriP*(US)-based ARS plasmids were unstable. However, parts of the P2 contents might be maintained via the reconstruction of a novel P2 plasmid together with novel genes in the surrounding environments (in mosaic of dark green and pink). In addition, the *oriP*(US) can be used to construct the novel plasmid (P3) with its surrounding genes (in pink).

It is known that some archaea use multiple origins to initiate chromosome replication, and these multiple-origin chromosomes are considered to originate from the integration of extrachromosomal elements (Robinson and Bell 2007). However, the detailed mechanisms, such as the evolutionary history of the development of multiple replication origins and the factors that govern the conserved or variable origins are unclear. Due to the broad conservation of *oriC1* across the archaeal domain of life, this origin was proposed to be present in the archaeal ancestral chromosome (Robinson et al. 2004; Wu et al. 2012). Importantly, replication-associated genes are clustered around *oriC1* in the characterized archaeal genomes (Myllykallio et al. 2000; Coker et al. 2009; Pelve et al. 2012). Furthermore, the genes around *oriC1* are highly conserved among haloarchaea (Coker et al. 2009). Thus, it is easy to conclude that the *oriC1* origin may be preserved from the archaeal ancestor via the conserved genomic environment in which replication-associated genes are abundant. Furthermore, we found that an rRNA operon was located in the *oriC1* origin in most haloarchaeal chromosomes, indicating that *oriC1* may have an effect on the expression and stability of surrounding genes. Thus, the location of *oriC1* may provide a selective advantage for the early replication and high expression of its neighboring replication-associated genes, which might sequentially promote the conservation of *oriC1* from the archaeal ancestral chromosome (fig. 6).

There are chromosomal replication origins that are only conserved in specific species, as illustrated by the conservation of *oriC2-cdc6E* in *Haloarcula* species. The origin *oriC2-cdc6E* was proposed to be acquired in the branch leading to the *Haloarcula* species and was stable via conservation. Unexpectedly, the deletion of *oriC2-cdc6E* showed no observable growth defects in *H. hispanica* (Wu et al. 2014), raising the question of what pressure was exerted to maintain this origin in *Haloarcula* species. Interestingly, the chromosomal location of *oriC2* is conserved, and another rRNA operon in the chromosome is located at a distance of only approximately 20 kb from *oriC2-cdc6E* (fig. 1*A*), suggesting that, in a similar manner as *oriC1*, this origin might be conserved via its effects on surrounding genes after its acquisition in the *Haloarcula* ancestor (fig. 6).

Apart from conserved replication origins, there are some variable origins in the chromosome that are not present in all strains, such as *oriC3-cdc6D* in the *H. hispanica* chromosome. Interestingly, these variable origins are normally located in chromosomal variable regions that are integrated at precisely the same position in each chromosome. We speculated that coupled insertion and deletion events occurred frequently in the chromosomal variable regions, resulting in the acquisition, loss, and disruption of replication origins in these regions (fig. 6). It was best to explain that replication origins in chromosomal variable regions are extremely diverse and that some origins are deficient. Taken together, we proposed that multiple-origin haloarchaeal chromosomes were developed in multiple steps, originating from the integration of replication origins into *oriC1*-dependent ancestral chromosome, and that the surrounding environments determined the fate of these novel origins, that is, conserved or variable (fig. 6).

Haloarchaeal genomes generally harbor extrachromosomal replicon(s), with as many as eight in *H. marismortui* (Baliga et al. 2004). Compared with the chromosome, extrachromosomal replicons are highly variable within specific species. Consistent with these findings, replication origins in these minireplicons are variable, which indicates that the frequent evolution of extrachromosomal elements might be associated with origin variation. In this study, based on the minichromosome and megaplasmid pHH400, we investigated the evolutionary association between extrachromosomal replicons and their bearing replication origins. We found that *oriC6*- and *oriC7*-based ARS plasmids are less stable compared with the *oriP* plasmid (fig. 3). In conjunction with the dynamics of the minichromosome and conservation of pHH400 in *Haloarcula* species, we speculated that the hierarchy of origins in plasmid stabilization might reflect the evolution of extrachromosomal replicons. Thus, we proposed a model of the evolutionary association between organization and replication origins of extrachromosomal replicons. In particular, similar to the *Haloarcula* minichromosome, P2 was unstable and was easily lost in the divergence of the species because the *oriP*(US)-based ARS plasmids were unstable. However, parts of the genes in P2 might be essential to cell viability; thus, these genes together with genes that promote adaptation to new environments appeared to be clustered in the reconstruction of a novel P2 (fig. 6). Conversely, the frequent reconstruction of P2 resulted in the variation of bearing replication origins. In addition, origins from P2 or from surrounding environments may be employed to construct novel minireplicons with surrounding genes (P3 in fig. 6), which would explain many strain-specific replicons. In contrast, similar to *Haloarcula* pHH400, P1 was stable in the divergence of species, as was the stability of the ARS plasmid with its bearing origin *oriP*(S) (fig. 6).

In conclusion, we have addressed the evolutionary association of multiple replication origins and multireplicon genome architecture, which includes evolution of multiple-origin chromosomes and evolution of organization and replication origins of extrachromosomal replicons. More interestingly, we suggested that this mechanism involved in the evolutionary association of multiple replication origins and multireplicon genome architecture is general in haloarchaea: 1) rRNA operon-mediated origin conservation appears to be universal in haloarchaea. Some sequenced haloarchaeal genomes have more than one rRNA operon in the chromosome (Hartman et al. 2010; Capes et al. 2011), and we previously suggested that the rRNA operon might benefit to the preservation of *oriCb* in *Haloferax volcanii*, *Halogeometricum borinquense*, and *Halorubrum lacusprofundi* from their ancestor (Wu et al. 2012) 2) The mechanism involved in the evolution of architecture and replication origins of extrachromosomal replicons is general in haloarchaea. For example, a comparison of megaplasmids between strain R1 and strain NRC-1 of *Halobacterium salinarum* revealed that these plasmids can rearrange even in the laboratory (Ng et al. 1998; Pfeiffer et al. 2008). We previously demonstrated that *Haloferax mediterranei* and *Haloferax volcanii* employed the same replication origin to construct completely different plasmids (Liu et al. 2013). In addition, it was also demonstrated that ARS plasmids based on replication origin of extrachromosomal replicon pHV1/4 are less stable than *oriC1* plasmids (Norais et al. 2007).

## Supplementary Material

Supplementary Data

## References

[evu219-B1] Altschul SF, Gish W, Miller W, Myers EW, Lipman DJ (1990). Basic local alignment search tool. J Mol Biol..

[evu219-B2] Bailey TL, Williams N, Misleh C, Li WW (2006). MEME: discovering and analyzing DNA and protein sequence motifs. Nucleic Acids Res..

[evu219-B3] Baliga NS (2004). Genome sequence of *Haloarcula marismortui*: a halophilic archaeon from the Dead Sea. Genome Res..

[evu219-B4] Berquist BR, Dassarma S (2003). An archaeal chromosomal autonomously replicating sequence element from an extreme halophile, *Halobacterium* sp. strain NRC-1. J Bacteriol..

[evu219-B5] Capes MD (2011). The information transfer system of halophilic archaea. Plasmid.

[evu219-B6] Cline SW, Lam WL, Charlebois RL, Schalkwyk LC, Doolittle WF (1989). Transformation methods for halophilic archaebacteria. Can J Microbiol..

[evu219-B7] Coker JA (2009). Multiple replication origins of *Halobacterium* sp. strain NRC-1:properties of the conserved *orc7*-dependent *oriC1*. J Bacteriol..

[evu219-B8] Di Rienzi SC (2012). Maintaining replication origins in the face of genomic change. Genome Res..

[evu219-B9] Ding JY, Chiang PW, Hong MJ, Dyall-Smith M, Tang SL (2014). Complete genome sequence of the extremely halophilic archaeon *Haloarcula hispanica* strain N61. Genome Announc..

[evu219-B10] Dueber EL, Corn JE, Bell SD, Berger JM (2007). Replication origin recognition and deformation by a heterodimeric archaeal Orc1 complex. Science.

[evu219-B11] Duggin IG, McCallum SA, Bell SD (2008). Chromosome replication dynamics in the archaeon *Sulfolobus acidocaldarius*. Proc Natl Acad Sci U S A..

[evu219-B12] Dyall-Smith ML (2011). *Haloquadratum walsbyi*: limited diversity in a global pond. PLoS One.

[evu219-B13] Grant JR, Stothard P (2008). The CGView Server: a comparative genomics tool for circular genomes. Nucleic Acids Res..

[evu219-B14] Guindon S (2010). New algorithms and methods to estimate maximum-likelihood phylogenies: assessing the performance of PhyML 3.0. Syst Biol..

[evu219-B15] Hartman AL (2010). The complete genome sequence of *Haloferax volcanii* DS2, a model archaeon. PLoS One.

[evu219-B16] Hawkins M, Malla S, Blythe MJ, Nieduszynski CA, Allers T (2013). Accelerated growth in the absence of DNA replication origins. Nature.

[evu219-B17] Liu H (2011). Complete genome sequence of *Haloarcula hispanica*, a model haloarchaeon for studying genetics, metabolism, and virus-host interaction. J Bacteriol..

[evu219-B18] Liu X (2013). Characterization of the minimal replicon of pHM300 and independent copy number control of major and minor chromosomes of *Haloferax mediterranei*. FEMS Microbiol Lett..

[evu219-B19] Lundgren M, Andersson A, Chen L, Nilsson P, Bernander R (2004). Three replication origins in *Sulfolobus* species: synchronous initiation of chromosome replication and asynchronous termination. Proc Natl Acad Sci U S A..

[evu219-B20] Lynch EA (2012). Sequencing of seven haloarchaeal genomes reveals patterns of genomic flux. PLoS One.

[evu219-B21] Matsunaga F, Forterre P, Ishino Y, Myllykallio H (2001). *In vivo* interactions of archaeal Cdc6/Orc1 and minichromosome maintenance proteins with the replication origin. Proc Natl Acad Sci U S A..

[evu219-B22] Matsunaga F, Norais C, Forterre P, Myllykallio H (2003). Identification of short ‘eukaryotic' Okazaki fragments synthesized from a prokaryotic replication origin. EMBO Rep..

[evu219-B23] Michel B, Bernander R (2014). Chromosome replication origins: do we really need them?. Bioessays.

[evu219-B24] Minegishi H (2010). Further refinement of the phylogeny of the *Halobacteriaceae* based on the full-length RNA polymerase subunit B' (*rpoB'*) gene. Int J Syst Evol Microbiol..

[evu219-B25] Muller CA, Nieduszynski CA (2012). Conservation of replication timing reveals global and local regulation of replication origin activity. Genome Res..

[evu219-B26] Myllykallio H (2000). Bacterial mode of replication with eukaryotic-like machinery in a hyperthermophilic archaeon. Science.

[evu219-B27] Ng WV (1998). Snapshot of a large dynamic replicon in a halophilic archaeon: megaplasmid or minichromosome?. Genome Res..

[evu219-B28] Norais C (2007). Genetic and physical mapping of DNA replication origins in *Haloferax volcanii*. PLoS Genet..

[evu219-B29] Paul S, Million-Weaver S, Chattopadhyay S, Sokurenko E, Merrikh H (2013). Accelerated gene evolution through replication-transcription conflicts. Nature.

[evu219-B30] Pelve EA, Lindas AC, Knoppel A, Mira A, Bernander R (2012). Four chromosome replication origins in the archaeon *Pyrobaculum calidifontis*. Mol Microbiol..

[evu219-B31] Pelve EA, Martens-Habbena W, Stahl DA, Bernander R (2013). Mapping of active replication origins in vivo in thaum- and euryarchaeal replicons. Mol Microbiol..

[evu219-B32] Pfeiffer F (2008). Evolution in the laboratory: the genome of *Halobacterium salinarum* strain R1 compared to that of strain NRC-1. Genomics.

[evu219-B33] Raymann K, Forterre P, Brochier-Armanet C, Gribaldo S (2014). Global phylogenomic analysis disentangles the complex evolutionary history of DNA replication in archaea. Genome Biol Evol..

[evu219-B34] Robinson NP, Bell SD (2005). Origins of DNA replication in the three domains of life. FEBS J..

[evu219-B35] Robinson NP, Bell SD (2007). Extrachromosomal element capture and the evolution of multiple replication origins in archaeal chromosomes. Proc Natl Acad Sci U S A..

[evu219-B36] Robinson NP (2004). Identification of two origins of replication in the single chromosome of the archaeon *Sulfolobus solfataricus*. Cell.

[evu219-B37] Samson RY (2013). Specificity and function of archaeal DNA replication initiator proteins. Cell Rep..

[evu219-B38] Wu Z, Liu H, Liu J, Liu X, Xiang H (2012). Diversity and evolution of multiple *orc/cdc6*-adjacent replication origins in haloarchaea. BMC Genomics.

[evu219-B39] Wu Z, Liu J, Yang H, Liu H, Xiang H (2014). Multiple replication origins with diverse control mechanisms in *Haloarcula hispanica*. Nucleic Acids Res..

[evu219-B40] Zhou L, Zhou M, Sun C, Xiang H, Tan H (2007). Genetic analysis of a novel plasmid pZMX101 from *Halorubrum saccharovorum*: determination of the minimal replicon and comparison with the related haloarchaeal plasmid pSCM201. FEMS Microbiol Lett..

