# Supplementary material for: Association between the Dynamics of Multiple Replication Origins and the Evolution of Multireplicon Genome Architecture in Haloarchaea
Source: Genome Biol Evol. 2014 Oct 3;6(10):2799–810. doi: 10.1093/gbe/evu219 (PMC4441112; doi:10.1093/gbe/evu219)
Supplement: Supplementary Data [file supp_evu219_suppl_data.zip › Table_S5.docx]

**Table S5. Percentage of missing data for each taxon in the concatenated alignment.**

| **Taxa** | **% missing data** |
| --- | --- |
| *Kluyveromyces lactis* | 58 |
| *Blastocystis sp.* | 43 |
| *Leucocryptos marina* | 38 |
| *Aurelia aurita* | 24 |
| *Hydra oligactis* | 14 |
| *Pavlova lutheri* | 12 |
| *Phaeocystis antarctica* | 12 |
| *Phaeocystis globosa* | 12 |
| *Phaeodactylum tricornutum* | 12 |
| *Ministeria vibrans* | 12 |
| *Nuclearia simplex* | 12 |
| *Gibberella moniliformis* | 12 |
| *Gigaspora rosea* | 12 |
| *Penicillium-digitatum* | 12 |
| *Allomyces macrogynus* | 12 |
| *Cyanidioschyzon merolae* | 12 |
| *Chondrus crispus* | 12 |
| *Emiliania huxleyi* | 12 |
| *Gracilariopsis andersonii* | 12 |
| *Monosiga brevocolis* | 12 |
| *Pyropia yezoensis* | 12 |
| *Cyanophora paradoxa* (NEIS 763) | 9 |
| *Pseudendoclonium akinetum* | 6 |
| *Nannochloropsis gaditana* | 2 |
| *Cyanophora biloba* (UTEX 2766) | 2 |
| *Acanthamoeba castellanii* | 1 |
| *Cyanophora tetracyanea* (NEIS 764) | 1 |
| *Dictyostelium discoideum* | 0 |
| *Bigelowiella natans* | 0 |
| *Glacuocystis nostochienarum (*UTEX 64) | 0 |
| *Chaetosphaeridium globosum* | 0 |
| *Pylaiella littoralis* | 0 |
| *Vermamoeba vermiformis* | 0 |
| *Chara vulgaris* | 0 |
| *Cyanophora paradoxa* (CCMP 329) | 0 |
| *Mesostigma viride* | 0 |
| *Chlorokybus atmophyticus* | 0 |
| *Coccomyxa sp.* | 0 |
| *Cyanoptyche gloeocystis* (SAG 4.97) | 0 |
| *Fucus vesiculosus* | 0 |
| *Gloeochaete wittrockiana* (SAG 46.84) | 0 |
| *Hemiselmis andersenii* | 0 |
| *Heterosigma akashiwo* | 0 |
| *Micromonas sp.* | 0 |
| *Nephroselmis olivacea* | 0 |
| *Phytophthora infestans* | 0 |
| *Pythium ultimum* | 0 |
| *Rhodomonas salina* | 0 |
| *Thalassiosira pseudonana* | 0 |
