# Supplementary material for: Association between the Dynamics of Multiple Replication Origins and the Evolution of Multireplicon Genome Architecture in Haloarchaea
Source: Genome Biol Evol. 2014 Oct 3;6(10):2799–810. doi: 10.1093/gbe/evu219 (PMC4441112; doi:10.1093/gbe/evu219)
Supplement: Supplementary Data [file supp_evu219_suppl_data.zip › Table_S6.docx]

**Table S6. Results of the Approximately Unbiased (AU) tests.** Alternative topologies not rejected by the test (p > 0.05) are highlighted in yellow.

| **Topology** | **AU p-value** |
| --- | --- |
| Fig. 3 Tree | 0.825 |
| Green algae with glaucophytes | 0.643 |
| Red algae with haptophytes | 0.116 |
| Glaucophytes with Stramenopiles | 0.078 |
| Glaucophytes with red algae | 0.077 |
| Haptophytes with red algae | 0.062 |
| Glaucophytes with Rhizarians | 0.055 |
| Glaucophytes with Katablepharids | 0.044 |
| Haptophytes with glaucophytes | 0.033 |
| Glaucophytes with haptophytes | 0.025 |
| Red algae with Cryptophytes | 0.019 |
| Haptophytes with green algae | 0.013 |
| Green algae with Stramenopiles | 0.01 |
| Red algae with Katablepharids | 0.006 |
| Green algae with haptophytes | 0.006 |
| Fungi with haptophytes | 0.005 |
| Glaucophytes with Cryptophytes | 0.004 |
| Red algae with Rhizarians | 0.003 |
| Green algae with Katablepharids | 0.003 |
| Glaucophytes with Amoeba | 0.002 |
| Green algae with Rhizarians | 0.002 |
| Green algae with Cryptophytes | 0.001 |
| Glaucophytes with Opisthokonts | 0.001 |
| *C. gloeocystis* with red algae | 0.001 |
| Green algae with Amoeba | 2.00E-04 |
| Red algae with Stramenopiles | 1.00E-04 |
| Green algae with Opisthokonts | 1.00E-04 |
| *C. gloeocystis* with green algae | 2.00E-05 |
| Red algae with Amoeba | 1.00E-06 |
| Fungi with Amoeba | 1.00E-07 |
| Red algae with Opisthokints | 1.00E-07 |
| Fungi with Rhizarians | 8.00E-08 |
| Red algae with *Emiliania* | 4.00E-14 |
| *Emiliania* with green algae | 2.00E-35 |
| *Emiliania* with red algae | 7.00E-39 |
| Fungi with Stramenopiles | 3.00E-46 |
| *Emiliania* with glaucophytes | 2.00E-54 |
| Fungi with glaucophytes | 6.00E-66 |
