# Supplementary material for: Association between the Dynamics of Multiple Replication Origins and the Evolution of Multireplicon Genome Architecture in Haloarchaea
Source: Genome Biol Evol. 2014 Oct 3;6(10):2799–810. doi: 10.1093/gbe/evu219 (PMC4441112; doi:10.1093/gbe/evu219)
Supplement: Supplementary Data [file supp_evu219_suppl_data.zip › Table_S7.docx]

**Table S7. Maximum likelihood bootstrap support for major clades, based on alignments of protein sequences (A) and nucleotide sequences (B) with progressive removal of fastest-evolving sites.**

**A.**

| **Alignment (% length)** | **Opisthokonts (% BS support)** | **Amoebozoa (% BS support)** | **Stramenopiles (% BS support)** | **Hacrobia (% BS support)** | **Archaeplastida (% BS support)** |
| --- | --- | --- | --- | --- | --- |
| 100% | 99 | 85 | 87 | 24 | 46 |
| 95% | 98 | 88 | 94 | 42 | 47 |
| 80% | 98 | 88 | 93 | N.A.^a^ | 35 |
| 70% | 98 | 84 | 83 | N.A.^b^ | 36 |

^a^ Katablepharids and Rhizaria branch together (55% BS) as sister to stramenopiles (41% BS). The haptophyte/cryptophyte clade receives 44% BS.

^b^ Katablepharids and Rhizaria branch together (47% BS) as sister to stramenopiles (28% BS). The haptophyte/cryptophyte clade receives 42% BS.

**B.**

| **Alignment (% length)** | **Opisthokonts (% BS support)** | **Amoebozoa (% BS support)** | **Stramenopiles (% BS support)** | **Hacrobia (% BS support)** | **Archaeplastida (% BS support)** |
| --- | --- | --- | --- | --- | --- |
| 100% | 92 | 68 | 100 | 69 | 51 |
| 95% | 92 | 68 | 100 | 74 | 52 |
| 80% | 96 | 68 | 100 | 79 | 47 |
| 70% | 94 | 56 | 100 | 82^a^ | N.A.^a^ |

^a^ Hacrobia branch within Archaeplastida as sister to red algae (44% BS for Hacrobia/red algae clade)
