# Supplementary material for: Association between the Dynamics of Multiple Replication Origins and the Evolution of Multireplicon Genome Architecture in Haloarchaea
Source: Genome Biol Evol. 2014 Oct 3;6(10):2799–810. doi: 10.1093/gbe/evu219 (PMC4441112; doi:10.1093/gbe/evu219)
Supplement: Supplementary Data [file supp_evu219_suppl_data.zip › Table_S8.docx]

**Table S8. Compositional homogeneity test for (A) the amino acid alignment and (B) the nucleotide alignment.** Significantly values of the Z-scores are marked with an asterix; these taxa exhibit compositional heterogenity that significantly violates assumptions of the model used (LGF and GTRI, respectively)

**A.**

|  | **Taxon** | **p-value** | **z-score** |
| --- | --- | --- | --- |
| * | *Acanthamoeba castellanii* | 0 | 9.893 |
|  | *Allomyces macrogynus* | 0.161 | 0.928 |
| * | *Aurelia aurita* | 0.008 | 3.351 |
| * | *Bigelowiella natans* | 0 | 9.565 |
| * | *Blastocystis sp.* | 0 | 7.848 |
| * | *Chaetosphaeridium globosum* | 0.001 | 6.157 |
| * | *Chara vulgaris* | 0.004 | 4.962 |
| * | *Chlorokybus atmophyticus* | 0.009 | 3.771 |
| * | *Chondrus crispus* | 0.001 | 4.84 |
| * | *Coccomyxa sp.* | 0.001 | 9.093 |
| * | *Cyanidioschyzon merolae* | 0 | 14.69 |
|  | *Cyanophora biloba* (UTEX 2766) | 0.091 | 1.491 |
| * | *Cyanophora paradoxa* (NEIS 763) | 0.01 | 3.395 |
| * | *Cyanophora paradoxa* (UTEX LB 555) | 0.003 | 3.64 |
| * | *Cyanophora tetracyanea* (NEIS 764) | 0.026 | 2.451 |
| * | *Cyanoptyche gloeocystis* (SAG 4.97) | 0 | 6.254 |
| * | *Dictyostelium discoideum* | 0 | 14.46 |
| * | *Emiliania huxleyi* | 0.003 | 3.576 |
| * | *Fucus vesiculosus* | 0.001 | 5.384 |
| * | *Gibberella moniliformis* | 0.026 | 2.394 |
| * | *Gigaspora rosea* | 0 | 14.468 |
| * | *Glacuocystis nostochienarum (*UTEX 64) | 0.033 | 2.183 |
| * | *Gloeochaete wittrockiana* (SAG 48.84) | 0.009 | 3.737 |
|  | *Gracilariopsis andersonii* | 0.221 | 0.631 |
|  | *Hemiselmis andersenii* | 0.418 | -0.003 |
| * | *Heterosigma akashiwo* | 0.002 | 8.682 |
| * | *Hydra oligactis* | 0 | 13.583 |
|  | *Kluyveromyces lactis* | 0.731 | -0.627 |
| * | *Leucocryptos marina* | 0.012 | 3.116 |
| * | *Mesostigma viride* | 0.001 | 7.29 |
| * | *Micromonas sp.* | 0.003 | 6.989 |
| * | *Ministeria vibrans* | 0 | 12.437 |
| * | *Monosiga brevocolis* | 0.02 | 2.809 |
| * | *Nannochloropsis gaditana* | 0.003 | 4.358 |
| * | *Nephroselmis olivacea* | 0.001 | 6.676 |
| * | *Nuclearia simplex* | 0 | 13.021 |
| * | *Pavlova lutheri* | 0.006 | 4.579 |
| * | *Penicillium digitatum* | 0.009 | 3.037 |
| * | *Phaeocystis antarctica* | 0 | 5.712 |
| * | *Phaeocystis globosa* | 0 | 7.913 |
|  | *Phaeodactylum tricornutum* | 0.101 | 1.295 |
| * | *Phytophthora infestans* | 0 | 7.523 |
| * | *Pseudendoclonium akinetum* | 0.001 | 8.61 |
| * | *Pylaiella littoralis* | 0 | 6.792 |
|  | *Pyropia yezoensis* | 0.448 | -0.005 |
| * | *Pythium ultimum* | 0.001 | 5.461 |
| * | *Rhodomonas salina* | 0 | 15.058 |
| * | *Thalassiosira pseudonana* | 0.039 | 2.182 |
| * | *Vermamoeba vermiformis* | 0.005 | 8.164 |
|  |  |  |  |
| * | Global test | 0 | 11.895 |

**B.**

|  | **Taxon** | **p-value** | **z-score** |
| --- | --- | --- | --- |
| * | *Acanthamoeba castellanii* | 0.017 | 2.468 |
| * | *Allomyces macrogynus* | 0.012 | 3.349 |
| * | *Aurelia aurita* | 0.009 | 4.885 |
| * | *Bigelowiella natans* | 0.015 | 5.672 |
| * | *Blastocystis sp.* | 0 | 19.24 |
|  | *Chaetosphaeridium globosum* | 0.222 | 0.185 |
| * | *Chara vulgaris* | 0.008 | 4.753 |
| * | *Chlorokybus atmophyticus* | 0.02 | 4.259 |
| * | *Chondrus crispus* | 0 | 10.749 |
| * | *Coccomyxa sp.* | 0 | 10.004 |
| * | *Cyanidioschyzon merolae* | 0 | 12.312 |
| * | *Cyanophora biloba* (UTEX 2766) | 0.019 | 3.241 |
| * | *Cyanophora paradoxa* (NEIS 763) | 0 | 6.692 |
| * | *Cyanophora paradoxa* (UTEX LB 555) | 0 | 8.503 |
| * | *Cyanophora tetracyanea* (NEIS 764) | 0 | 8.25 |
| * | *Cyanoptyche gloeocystis* (SAG 4.97) | 0 | 13.361 |
| * | *Dictyostelium discoideum* | 0 | 15.521 |
| * | *Emiliania huxleyi* | 0.017 | 3.081 |
| * | *Fucus vesiculosus* | 0.006 | 6.998 |
| * | *Gibberella moniliformis* | 0 | 20.263 |
| * | *Gigaspora rosea* | 0.011 | 7.528 |
| * | *Glacuocystis nostochienarum (*UTEX 64) | 0 | 11.36 |
| * | *Gloeochaete wittrockiana* (SAG 48.84) | 0.011 | 7.337 |
| * | *Gracilariopsis andersonii* | 0 | 6.957 |
|  | *Hemiselmis andersenii* | 0.121 | 0.725 |
| * | *Heterosigma akashiwo* | 0.006 | 9.745 |
| * | *Hydra oligactis* | 0 | 36.082 |
| * | *Kluyveromyces lactis* | 0 | 10.552 |
|  | *Leucocryptos marina* | 0.72 | -0.648 |
|  | *Mesostigma viride* | 0.099 | 1.251 |
| * | *Micromonas sp.* | 0.015 | 3.516 |
| * | *Ministeria vibrans* | 0 | 19.978 |
| * | *Monosiga brevocolis* | 0 | 25.34 |
| * | *Nannochloropsis gaditana* | 0.011 | 3.77 |
|  | *Nephroselmis olivacea* | 0.321 | -0.047 |
| * | *Nuclearia simplex* | 0 | 22.142 |
| * | *Pavlova lutheri* | 0.004 | 13.179 |
| * | *Penicillium digitatum* | 0 | 26.569 |
| * | *Phaeocystis antarctica* | 0.009 | 6.471 |
| * | *Phaeodactylum tricornutum* | 0.007 | 3.712 |
| * | *Phaeocystis globosa* | 0.01 | 6.191 |
| * | *Phytophthora infestans* | 0 | 24.073 |
| * | *Pseudendoclonium akinetum* | 0.012 | 4.767 |
| * | *Pylaiella littoralis* | 0.009 | 6.787 |
| * | *Pyropia yezoensis* | 0.043 | 1.627 |
| * | *Pythium ultimum* | 0 | 16.808 |
| * | *Rhodomonas salina* | 0.01 | 5.458 |
|  | *Thalassiosira pseudonana* | 0.47 | -0.269 |
| * | *Vermamoeba vermiformis* | 0.013 | 5.382 |
|  |  |  |  |
| * | Global test | 0 | 7.933 |
