# Supplementary material for: Association between the Dynamics of Multiple Replication Origins and the Evolution of Multireplicon Genome Architecture in Haloarchaea
Source: Genome Biol Evol. 2014 Oct 3;6(10):2799–810. doi: 10.1093/gbe/evu219 (PMC4441112; doi:10.1093/gbe/evu219)
Supplement: Supplementary Data [file supp_evu219_suppl_data.zip › Table_S9.docx]

**Table S9. Codon usage of glaucophyte mtDNA canonical genes.** (A) Frequency of each codon type, normalised per 1000 nucleotides. (B) Graph of codon type plotted against frequency, normalised per 1000 nucleotides. *Cg*: *Cyanoptyche gloeocystis; Gw*: *Gloeochaete wittrockiana*; *Cp*: *Cyanophora paradoxa*; *Gn*: *Glaucocystis nostochinearum*

**A.**

| **Amino Acid** | **Codon** | ***Cg* /1000** | ***Gw* /1000** | ***Cp* /1000** | ***Gn* /1000** |
| --- | --- | --- | --- | --- | --- |
| **Ala** | GCG | 3.98 | 5.29 | 4.01 | 5.29 |
| **Ala** | GCA | 18.89 | 14.07 | 20.44 | 16.96 |
| **Ala** | GCT | 18.64 | 29.69 | 17.15 | 20.09 |
| **Ala** | GCC | 3.21 | 1.81 | 2.80 | 2.17 |
| **Cys** | TGT | 5.53 | 6.45 | 9.00 | 6.50 |
| **Cys** | TGC | 5.14 | 2.19 | 1.46 | 1.80 |
| **Asp** | GAT | 21.72 | 24.01 | 19.46 | 22.85 |
| **Asp** | GAC | 4.24 | 4.26 | 2.80 | 2.77 |
| **Glu** | GAG | 6.81 | 8.65 | 4.38 | 4.69 |
| **Glu** | GAA | 22.11 | 21.82 | 23.24 | 26.46 |
| **Phe** | TTT | 62.85 | 79.65 | 68.73 | 82.39 |
| **Phe** | TTC | 11.83 | 8.78 | 9.85 | 6.98 |
| **Gly** | GGG | 7.84 | 6.71 | 6.08 | 4.69 |
| **Gly** | GGA | 14.14 | 13.94 | 15.69 | 22.49 |
| **Gly** | GGT | 22.62 | 36.66 | 27.74 | 21.77 |
| **Gly** | GGC | 6.30 | 3.74 | 4.01 | 3.37 |
| **His** | CAT | 13.11 | 16.91 | 17.15 | 16.72 |
| **His** | CAC | 4.37 | 2.19 | 2.43 | 1.68 |
| **Ile** | ATA | 70.18 | 35.50 | 49.51 | 46.79 |
| **Ile** | ATT | 45.50 | 48.67 | 66.55 | 60.98 |
| **Ile** | ATC | 9.00 | 7.23 | 7.18 | 6.98 |
| **Lys** | AAG | 14.01 | 12.65 | 5.23 | 5.89 |
| **Lys** | AAA | 68.89 | 49.57 | 78.47 | 74.09 |
| **Leu** | TTG | 12.60 | 34.47 | 12.17 | 15.76 |
| **Leu** | TTA | 71.08 | 66.49 | 85.64 | 100.91 |
| **Leu** | CTG | 4.24 | 3.74 | 1.70 | 1.20 |
| **Leu** | CTA | 19.41 | 8.39 | 11.19 | 7.46 |
| **Leu** | CTT | 12.60 | 15.49 | 13.50 | 11.31 |
| **Leu** | CTC | 2.83 | 0.90 | 0.61 | 0.48 |
| **Met** | ATG | 23.78 | 26.85 | 24.09 | 20.69 |
| **Asn** | AAT | 50.13 | 38.34 | 57.66 | 57.73 |
| **Asn** | AAC | 16.71 | 8.13 | 8.64 | 8.18 |
| **Pro** | CCG | 3.73 | 4.13 | 2.80 | 3.25 |
| **Pro** | CCA | 12.47 | 11.23 | 14.11 | 13.47 |
| **Pro** | CCT | 12.21 | 16.65 | 11.44 | 12.39 |
| **Pro** | CCC | 0.64 | 1.94 | 1.95 | 1.68 |
| **Gln** | CAG | 4.76 | 7.49 | 3.41 | 3.13 |
| **Gln** | CAA | 18.77 | 17.43 | 22.02 | 19.85 |
| **Arg** | AGG | 3.86 | 2.84 | 1.82 | 1.32 |
| **Arg** | AGA | 16.97 | 11.36 | 12.41 | 11.19 |
| **Arg** | CGG | 0.13 | 0.39 | 0.00 | 0.60 |
| **Arg** | CGA | 2.57 | 4.13 | 4.99 | 5.17 |
| **Arg** | CGT | 2.70 | 11.62 | 12.29 | 8.30 |
| **Arg** | CGC | 0.77 | 2.07 | 1.70 | 1.32 |
| **Ser** | AGT | 17.10 | 20.53 | 17.15 | 19.36 |
| **Ser** | AGC | 10.28 | 4.78 | 4.38 | 3.13 |
| **Ser** | TCG | 3.98 | 6.58 | 3.41 | 5.29 |
| **Ser** | TCA | 21.59 | 17.30 | 21.65 | 19.12 |
| **Ser** | TCT | 17.22 | 25.17 | 17.15 | 21.05 |
| **Ser** | TCC | 3.34 | 3.49 | 3.16 | 1.80 |
| **Thr** | ACG | 3.98 | 3.61 | 1.95 | 3.13 |
| **Thr** | ACA | 24.42 | 17.94 | 24.21 | 20.45 |
| **Thr** | ACT | 19.41 | 23.75 | 17.15 | 18.88 |
| **Thr** | ACC | 4.11 | 3.61 | 3.77 | 2.77 |
| **Val** | GTG | 6.56 | 8.13 | 3.04 | 2.29 |
| **Val** | GTA | 22.24 | 21.56 | 19.10 | 15.16 |
| **Val** | GTT | 21.08 | 40.80 | 25.91 | 25.14 |
| **Val** | GTC | 2.83 | 3.49 | 1.46 | 2.77 |
| **Trp** | TGG | 12.98 | 13.94 | 13.14 | 14.43 |
| **Tyr** | TAT | 37.40 | 40.54 | 46.11 | 45.59 |
| **Tyr** | TAC | 13.37 | 5.94 | 5.60 | 5.77 |
| **Stop** | TGA | 0.51 | 0.13 | 0.00 | 0.00 |
| **Stop** | TAG | 0.39 | 0.39 | 0.24 | 0.72 |
| **Stop** | TAA | 3.34 | 3.74 | 3.89 | 3.37 |

**B.**

**
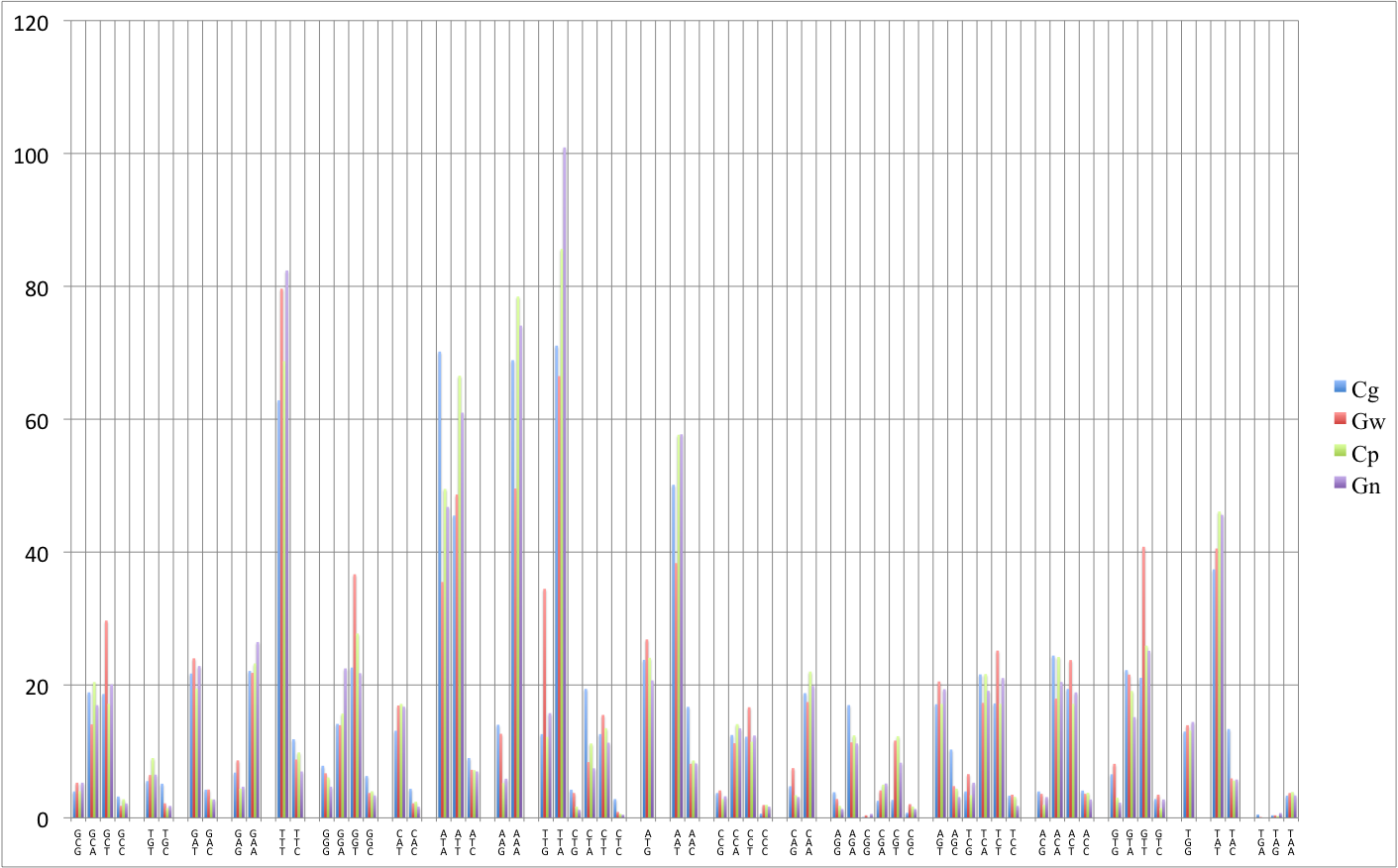
**
