# Supplementary material for: Association between the Dynamics of Multiple Replication Origins and the Evolution of Multireplicon Genome Architecture in Haloarchaea
Source: Genome Biol Evol. 2014 Oct 3;6(10):2799–810. doi: 10.1093/gbe/evu219 (PMC4441112; doi:10.1093/gbe/evu219)
Supplement: Supplementary Data [file supp_evu219_suppl_data.zip › Table_S1.docx]

**Table S1. Glaucophyte mtDNA unidentified ORF (uORF) statistics.** For *C. paradoxa* and *G. nostochinearum*, uORF names in parentheses correspond to ORF annotations in Genbank accessions NC_017836 and NC_015117, respectively.

|  | **Length (bp)** | **Start  codon** | **Stop  codon** | **A+T  content** | **Mean/min/max % A+T content canonical genes** | **% A+T content intergenic  regions^a^** | **Mean/min/max length (bp) of  canonical genes** |
| --- | --- | --- | --- | --- | --- | --- | --- |
| ***C. gloeocystis*** |  |  |  |  |  |  |  |
| uORF-A | 312 | AUG | UAA | 76.9 | 72.3/68.4/79.2 | 83.8/79.1 | 707/219/2022 |
| uORF-B | 570 | AUG | UAG | 71.4 |  |  |  |
| uORF-C | 909 | AUG | UAA | 80.9 |  |  |  |
| uORF-D | 279 | AUG | UGA | 77.4 |  |  |  |
| uORF-E | 222 | AUG | UAG | 86.9 |  |  |  |
| uORF-F | 294 | AUG | UAA | 71.8 |  |  |  |
| ***G. wittrockiana*** |  |  |  |  |  |  |  |
| uORF-A | 279 | UUG | UAA | 71.3 | 69.7/66/75.9 | 79.3/76.5 | 704/192/2022 |
| uORF-B | 312 | AUG | UAG | 68.3 |  |  |  |
| uORF-C | 411 | AUG | UAA | 75.2 |  |  |  |
| uORF-D | 480 | AUG | UAG | 74.4 |  |  |  |
| uORF-E | 645 | AUG | UGA | 71.2 |  |  |  |
| uORF-F | 402 | AUG | UAA | 69.0 |  |  |  |
| ***C. paradoxa*** |  |  |  |  |  |  |  |
| uORF-A (orf162) | 489 | AUG | UAA | 85.3 | 74.4/67.1/84.9 | 80.8/78.7 | 726/225/1998 |
| uORF-B (orf166) | 501 | AUG | UGA | 71.1 |  |  |  |
| uORF-C (orf181) | 546 | AUG | UAA | 86.6 |  |  |  |
| uORF-D (orf229 2) | 690 | AUG | UAA | 80.7 |  |  |  |
| uORF-E (orf229) | 690 | AUG | UAA | 71.7 |  |  |  |
| uORF-F (orf344) | 1035 | AUG | UAA | 83.8 |  |  |  |
| uORF-G (orf427) | 1284 | AUG | UAA | 78.7 |  |  |  |
| uORF-H (orf533) | 1602 | AUG | UAA | 75.5 |  |  |  |
| uORF-I (orf535) | 1608 | AUG | UAA | 70.6 |  |  |  |
| uORF-J (orf544) | 1635 | AUG | UAA | 76.8 |  |  |  |
| ***G. nostochinearum*** |  |  |  |  |  |  |  |
| uORF-A (orf164) | 495 | AUG | UAA | 83.8 | 75.1/67.6/86.5 | 85.6/85 | 733/225/1992 |
| uORF-B (orf211) | 636 | AUG | UGA | 82.2 |  |  |  |

^a^ Values including unidentified ORFs as bonafide genes / values not including unidentified ORFs as bonafide genes
