# Supplementary material for: Association between the Dynamics of Multiple Replication Origins and the Evolution of Multireplicon Genome Architecture in Haloarchaea
Source: Genome Biol Evol. 2014 Oct 3;6(10):2799–810. doi: 10.1093/gbe/evu219 (PMC4441112; doi:10.1093/gbe/evu219)
Supplement: Supplementary Data [file supp_evu219_suppl_data.zip › Table_S2.docx]

**Table S2. Amino acid compositions of glaucophyte mtDNA unidentified ORFs.** For *C. paradoxa* and *G. nostochinearum*, see Table S1 for uORF annotations corresponding to Genbank accessions NC_017836 and NC_015117, respectively.

|  | **Amino  acid** | **Average  % in canonical genes** | **% in  each  uORF** |  |  |  |  |  |  |  |  |  |
| --- | --- | --- | --- | --- | --- | --- | --- | --- | --- | --- | --- | --- |
| ***C. paradoxa*** |  |  | **uORF-A** | **uORF-B** | **uORF-C** | **uORF-D** | **uORF-E** | **uORF-F** | **uORF-G** | **uORF-H** | **uORF-I** | **uORF-J** |
|  | A | 4.5 | 0.0 | 3.0 | 0.6 | 3.1 | 1.3 | 0.3 | 0.9 | 1.9 | 3.2 | 1.8 |
|  | C | 1.0 | 0.6 | 1.8 | 1.1 | 1.7 | 1.7 | 1.5 | 2.1 | 1.1 | 1.7 | 1.1 |
|  | D | 2.1 | 1.9 | 3.6 | 1.7 | 5.7 | 1.7 | 2.0 | 5.4 | 4.9 | 2.6 | 4.2 |
|  | E | 2.8 | 2.5 | 6.0 | 1.1 | 7.0 | 3.5 | 3.2 | 4.0 | 3.0 | 4.1 | 2.2 |
|  | F | 8.0 | 12.3 | 9.6 | 9.4 | 9.6 | 14.0 | 13.4 | 9.6 | 6.9 | 10.1 | 7.0 |
|  | G | 5.5 | 1.2 | 2.4 | 1.1 | 3.1 | 1.3 | 1.2 | 2.6 | 2.1 | 2.4 | 2.2 |
|  | H | 2.0 | 1.2 | 1.8 | 1.1 | 1.3 | 1.3 | 1.7 | 1.2 | 2.4 | 1.1 | 2.6 |
|  | I | 12.4 | 11.1 | 9.0 | 11.0 | 8.7 | 17.9 | 16.0 | 11.9 | 10.3 | 9.7 | 12.1 |
|  | K | 8.4 | 17.9 | 9.6 | 14.9 | 5.7 | 7.0 | 6.1 | 10.8 | 11.8 | 11.4 | 11.8 |
|  | L | 12.5 | 10.5 | 13.3 | 12.2 | 13.5 | 11.8 | 15.4 | 13.1 | 13.5 | 9.5 | 15.4 |
|  | M | 2.5 | 1.2 | 1.8 | 1.7 | 3.5 | 0.4 | 1.7 | 1.6 | 2.6 | 2.6 | 2.4 |
|  | N | 6.3 | 13.6 | 5.4 | 18.2 | 7.4 | 7.0 | 10.8 | 9.1 | 7.3 | 2.8 | 8.5 |
|  | P | 3.0 | 1.2 | 2.4 | 0.6 | 0.9 | 1.7 | 0.9 | 2.1 | 3.4 | 3.9 | 2.8 |
|  | Q | 2.5 | 1.9 | 1.8 | 4.4 | 4.4 | 1.7 | 2.3 | 2.8 | 4.5 | 3.2 | 4.0 |
|  | R | 3.3 | 3.1 | 5.4 | 0.6 | 4.4 | 2.6 | 2.3 | 2.6 | 1.7 | 4.9 | 1.7 |
|  | S | 6.6 | 6.8 | 5.4 | 3.9 | 3.9 | 6.1 | 6.7 | 6.1 | 9.2 | 5.4 | 8.3 |
|  | T | 4.7 | 4.3 | 4.8 | 7.2 | 4.4 | 4.8 | 1.7 | 4.2 | 5.4 | 3.2 | 4.4 |
|  | V | 5.1 | 1.9 | 3.6 | 2.2 | 3.5 | 5.2 | 2.9 | 2.8 | 4.1 | 6.2 | 3.7 |
|  | W | 1.3 | 0.0 | 3.6 | 0.0 | 1.7 | 0.0 | 0.3 | 0.2 | 0.4 | 3.9 | 0.4 |
|  | Y | 5.2 | 6.8 | 5.4 | 7.2 | 6.6 | 8.7 | 9.6 | 6.8 | 3.4 | 8.0 | 3.5 |
| ***G. nostochinearum*** |  |  | **uORF-A** | **uORF-B** |  |  |  |  |  |  |  |  |
|  | A | 4.5 | 0.6 | 0.9 |  |  |  |  |  |  |  |  |
|  | C | 0.8 | 0.6 | 0.0 |  |  |  |  |  |  |  |  |
|  | D | 2.6 | 1.2 | 3.8 |  |  |  |  |  |  |  |  |
|  | E | 3.1 | 2.4 | 2.4 |  |  |  |  |  |  |  |  |
|  | F | 8.9 | 8.5 | 14.2 |  |  |  |  |  |  |  |  |
|  | G | 5.2 | 2.4 | 2.4 |  |  |  |  |  |  |  |  |
|  | H | 1.8 | 2.4 | 0.9 |  |  |  |  |  |  |  |  |
|  | I | 11.5 | 10.9 | 9.9 |  |  |  |  |  |  |  |  |
|  | K | 8.0 | 14.5 | 12.7 |  |  |  |  |  |  |  |  |
|  | L | 13.7 | 16.4 | 12.7 |  |  |  |  |  |  |  |  |
|  | M | 2.1 | 1.2 | 1.9 |  |  |  |  |  |  |  |  |
|  | N | 6.6 | 14.5 | 10.8 |  |  |  |  |  |  |  |  |
|  | P | 3.1 | 1.2 | 0.9 |  |  |  |  |  |  |  |  |
|  | Q | 2.3 | 0.6 | 1.4 |  |  |  |  |  |  |  |  |
|  | R | 2.8 | 1.2 | 3.8 |  |  |  |  |  |  |  |  |
|  | S | 7.0 | 7.9 | 7.5 |  |  |  |  |  |  |  |  |
|  | T | 4.5 | 1.2 | 4.7 |  |  |  |  |  |  |  |  |
|  | V | 4.5 | 6.1 | 1.9 |  |  |  |  |  |  |  |  |
|  | W | 1.4 | 0.6 | 0.0 |  |  |  |  |  |  |  |  |
|  | Y | 5.1 | 4.8 | 6.6 |  |  |  |  |  |  |  |  |
| ***G. wittrockiana*** |  |  | **uORF-A** | **uORF-B** | **uORF-C** | **uORF-D** | **uORF-E** | **uORF-F** |  |  |  |  |
|  | A | 5.1 | 1.1 | 6.7 | 3.0 | 1.3 | 1.9 | 3.7 |  |  |  |  |
|  | C | 0.9 | 2.2 | 1.0 | 0.6 | 1.3 | 0.9 | 0.7 |  |  |  |  |
|  | D | 2.8 | 3.2 | 3.8 | 4.3 | 3.1 | 7.9 | 4.5 |  |  |  |  |
|  | E | 3.0 | 4.3 | 13.5 | 2.4 | 1.3 | 5.6 | 5.2 |  |  |  |  |
|  | F | 8.8 | 11.8 | 4.8 | 9.1 | 9.4 | 7.4 | 6.0 |  |  |  |  |
|  | G | 6.1 | 3.2 | 1.0 | 1.2 | 5.6 | 6.0 | 5.2 |  |  |  |  |
|  | H | 1.9 | 2.2 | 1.9 | 1.8 | 3.1 | 1.4 | 4.5 |  |  |  |  |
|  | I | 9.1 | 10.8 | 10.6 | 11.0 | 9.4 | 10.2 | 9.0 |  |  |  |  |
|  | K | 6.2 | 8.6 | 13.5 | 6.7 | 10.6 | 7.4 | 7.5 |  |  |  |  |
|  | L | 12.9 | 15.1 | 10.6 | 17.7 | 16.3 | 11.2 | 9.7 |  |  |  |  |
|  | M | 2.7 | 2.2 | 3.8 | 3.0 | 1.3 | 2.8 | 0.7 |  |  |  |  |
|  | N | 4.6 | 5.4 | 2.9 | 4.9 | 5.6 | 5.1 | 8.2 |  |  |  |  |
|  | P | 3.4 | 3.2 | 1.0 | 0.6 | 1.3 | 2.3 | 5.2 |  |  |  |  |
|  | Q | 2.5 | 1.1 | 5.8 | 3.0 | 2.5 | 3.3 | 5.2 |  |  |  |  |
|  | R | 3.2 | 1.1 | 3.8 | 1.8 | 1.3 | 3.7 | 3.0 |  |  |  |  |
|  | S | 7.8 | 4.3 | 3.8 | 8.5 | 13.8 | 5.6 | 4.5 |  |  |  |  |
|  | T | 4.9 | 6.5 | 5.8 | 6.1 | 4.4 | 5.6 | 5.2 |  |  |  |  |
|  | V | 7.4 | 5.4 | 2.9 | 6.1 | 4.4 | 4.7 | 4.5 |  |  |  |  |
|  | W | 1.4 | 0.0 | 1.0 | 0.6 | 0.0 | 0.5 | 0.0 |  |  |  |  |
|  | Y | 4.6 | 7.5 | 1.0 | 6.7 | 3.8 | 6.0 | 6.7 |  |  |  |  |
| ***C. gloeocystis*** |  |  | **uORF-A** | **uORF-B** | **uORF-C** | **uORF-D** | **uORF-E** | **uORF-F** |  |  |  |  |
|  | A | 4.5 | 1.0 | 4.8 | 1.3 | 4.3 | 2.7 | 1.0 |  |  |  |  |
|  | C | 1.1 | 2.9 | 2.1 | 1.3 | 1.1 | 1.4 | 1.0 |  |  |  |  |
|  | D | 2.6 | 1.9 | 5.3 | 2.0 | 1.1 | 2.7 | 14.4 |  |  |  |  |
|  | E | 2.9 | 0.0 | 5.3 | 1.7 | 5.4 | 1.4 | 7.2 |  |  |  |  |
|  | F | 7.5 | 2.9 | 10.6 | 7.9 | 12.0 | 11.0 | 6.2 |  |  |  |  |
|  | G | 5.1 | 4.9 | 2.1 | 0.7 | 2.2 | 1.4 | 5.2 |  |  |  |  |
|  | H | 1.7 | 2.9 | 1.6 | 1.7 | 0.0 | 1.4 | 2.1 |  |  |  |  |
|  | I | 12.5 | 10.7 | 15.3 | 8.6 | 17.4 | 9.6 | 9.3 |  |  |  |  |
|  | K | 8.3 | 13.6 | 3.2 | 18.9 | 7.6 | 27.4 | 6.2 |  |  |  |  |
|  | L | 12.3 | 13.6 | 14.3 | 12.9 | 13.0 | 5.5 | 7.2 |  |  |  |  |
|  | M | 2.4 | 1.0 | 1.6 | 1.3 | 4.3 | 2.7 | 6.2 |  |  |  |  |
|  | N | 6.7 | 13.6 | 5.3 | 15.2 | 7.6 | 12.3 | 2.1 |  |  |  |  |
|  | P | 2.9 | 1.0 | 1.6 | 0.3 | 1.1 | 0.0 | 0.0 |  |  |  |  |
|  | Q | 2.4 | 1.9 | 3.2 | 2.6 | 0.0 | 2.7 | 1.0 |  |  |  |  |
|  | R | 2.7 | 1.0 | 2.1 | 4.0 | 3.3 | 0.0 | 2.1 |  |  |  |  |
|  | S | 7.4 | 8.7 | 5.3 | 6.6 | 3.3 | 1.4 | 6.2 |  |  |  |  |
|  | T | 5.2 | 5.8 | 2.6 | 5.0 | 5.4 | 1.4 | 6.2 |  |  |  |  |
|  | V | 5.3 | 3.9 | 9.0 | 2.3 | 4.3 | 2.7 | 7.2 |  |  |  |  |
|  | W | 1.3 | 0.0 | 0.0 | 1.0 | 1.1 | 0.0 | 2.1 |  |  |  |  |
|  | Y | 5.1 | 8.7 | 4.8 | 4.6 | 5.4 | 12.3 | 7.2 |  |  |  |  |
