# Supplementary material for: Association between the Dynamics of Multiple Replication Origins and the Evolution of Multireplicon Genome Architecture in Haloarchaea
Source: Genome Biol Evol. 2014 Oct 3;6(10):2799–810. doi: 10.1093/gbe/evu219 (PMC4441112; doi:10.1093/gbe/evu219)
Supplement: Supplementary Data [file supp_evu219_suppl_data.zip › Table_S3.docx]

**Table S3. Characteristics of glaucophyte mtDNA canonical protein-coding genes.**

| **Gene** | **Start  codon** | **Stop  codon** | **% A+T  content Cp/Gn/Gw/Cg** |
| --- | --- | --- | --- |
| *atp4* | AUG | UAA | 77.7 / 83.5 / 77.8 / 78.6 |
| *atp6* | AUG | UAA (Cg=UAG) | 72.6 / 74.5 / 70.5 / 70.7 |
| *atp8* | AUG | UAA | 80.7 / 78.6 / 73.0 / 80.6 |
| *atp9* | AUG | UAA | 68.9 / 68.0 / 65.3 / 66.2 |
| *cob* | AUG | UAA | 70.7 / 98.7 / 66.9 / 68.5 |
| *cox1* | AUG | UAA | 67.2 / 66.4 / 66.7 / 66.2 |
| *cox2* | AUG | UAA  (Gw=UAG) | 68.1 / 72.3 / 65.1 / 68.4 |
| *cox3* | AUG | UAA (Gn=UAG) | 67.1 / 67.6 / 66.0 / 68.4 |
| *nad1* | AUG | UAA (Cg=UGA) | 69.5 / 68.7 / 66.1 / 67.7 |
| *nad11* | AUG | UAA (Cg=UGA) | 77.1 / 76.9 / 68.4 / 74.7 |
| *nad2* | AUG | UAA | 78.0 / 77.0 / 70.8 / 73.8 |
| *nad3* | AUG  (Cg=UUG) | UAA | 74.5 / 72.0 / 69.1 / 71.8 |
| *nad4* | AUG | UAA | 73.8 / 74.2 / 67.9 / 70.8 |
| *nad4L* | AUG | UAA | 75.7 / 73.1 / 71.8 / 75.1 |
| *nad5* | AUG | UAA (Cp=UAG) | 72.5 / 73.6 / 67.9 / 69.9 |
| *nad6* | AUG | UAA (Cg=UAG) | 81.3 / 80.4 / 73.4 / 75.3 |
| *nad7* | AUG | UAA | 68.9 / 66.8 / 64.7 / 67.7 |
| *nad9* | AUG | UAA | 74.0 / 74.1 / 72.0 / 69.3 |
| *rpl14* | AUG | UAA (Gn=UAG) | 77.2 / 78.5 / 69.2 / 77.1 |
| *rpl16* | AUG | AUG (Cp=UAG) | 67.6 / 75.5 / 73.5 / 73.3 |
| *rpl2* | AUG | UAA (Gw=UAG) | 72.3 / 72.8 / 69.1 / 70.2 |
| *rpl5* | AUG | UAA (Cg=UGA) | 78.6 / 82.6 / N.A. / 77.1 |
| *rpl6* | AUG | UAA | 83.8 / 84.4 / 69.3 / 79.2 |
| *rps10* | AUG | UAA | 80.6 / 82.2 / 76.5 / 82.8 |
| *rps11* | AUG | UAA | 78.5 / 74.6 / 71.1 / 77.0 |
| *rps12* | AUG | UAA | 67.2 / 73.5 / 66.7 / 68.9 |
| *rps13* | AUG | UAA | 74.7 / 76.6 / 73.2 / 76.3 |
| *rps14* | AUG | UAA (Gn=UAG) (Gw=UAG) | 79.0 / 80.2 / 70.0 / 81.1 |
| *rps3* | AUG | UAA (Gn=UAG) | 77.7 / 81.7 / 77.6 / 78.8 |
| *rps4* | AUG | UAA (Gn=UAG) | 84.9 / 86.5 / 75.9 / N.A. |
| *rps7* | AUG | UAA (Gn=UAG)  (Gw=UGA) (Cg=UAG) | 77.9 / 82.3 / 78.6 / 78.6 |
| *rps19* | AUG | UAA | 76.0 / 80.7 / 77.1 / 79.9 |
| *sdh3* | AUG | UAA (Cg=UGA) | 79.8 / 81.4 / 76.2 / 76.4 |
| *sdh4* | AUG | UAA | 80.4 / 81.9 / 77.4 / 73.5 |

Cp: *Cyanophora paradoxa*; Gn: *Glaucocystis nostochinearum*; Cg: *Cyanoptyche gloeocystis*; Gw: *Gloeochaete wittrockiana*
