# Supplementary material for: Association between the Dynamics of Multiple Replication Origins and the Evolution of Multireplicon Genome Architecture in Haloarchaea
Source: Genome Biol Evol. 2014 Oct 3;6(10):2799–810. doi: 10.1093/gbe/evu219 (PMC4441112; doi:10.1093/gbe/evu219)
Supplement: Supplementary Data [file supp_evu219_suppl_data.zip › Table_S4.docx]

**Table S4. Taxa present in original multiple alignment.** Excavates and Alveolates were removed from later analyses.

| **Group** | **Taxa** |
| --- | --- |
| Glaucophytes | *Cyanoptyche gloeocystis* (SAG 4.97) |
|  | *Gloeochaete wittrockiana* (SAG 48.84) |
|  | *Glacuocystis nostochinearum (*UTEX 64) |
|  | *Cyanophora paradoxa* (NEIS 763) |
|  | *Cyanophora tetracyanea* (NEIS 764) |
|  | *Cyanophora biloba* (UTEX 2766) |
|  | *Cyanophora paradoxa* (CCMP 329) |
| Rhodophyta | *Chondrus crispus* |
|  | *Cyanidioschyzon merolae* |
|  | *Gracilariopsis andersonii* |
|  | *Pyropia yezoensis* |
| Viridiplantae | *Chaetosphaeridium globosum* |
|  | *Chara vulgaris* |
|  | *Chlorokybus atmophyticus* |
|  | *Coccomyxa sp.* |
|  | *Mesostigma viride* |
|  | *Micromonas sp.* |
|  | *Nephroselmis olivacea* |
|  | *Pseudendoclonium akinetum* |
| Opisthokonts | *Allomyces macrogynus* |
|  | *Aurelia aurita* |
|  | *Gibberella moniliformis* |
|  | *Gigaspora rosea* |
|  | *Ministeria vibrans* |
|  | *Monosiga brevicollis* |
|  | *Nuclearia simplex* |
|  | *Penicillium digitatum* |
|  | *Hydra oligactis* |
|  | *Kluyveromyces lactis* |
| Amoebozoa | *Acanthamoeba castellanii* |
|  | *Dictyostelium discoideum* |
|  | *Vermamoeba vermiformis* |
| Stramenopiles | *Fucus vesiculosus* |
|  | *Nannochloropsis gaditana* |
|  | *Phaeodactylum tricornutum* |
|  | *Phytophthora infestans* |
|  | *Pylaiella littoralis* |
|  | *Pythium ultimum* |
|  | *Thalassiosira pseudonana* |
|  | *Heterosigma akashiwo* |
|  | *Blastocystis sp.* |
| Cryptophytes | *Hemiselmis andersenii* |
|  | *Rhodomonas salina* |
| Katablepharids | *Leucocryptos marina* |
| Haptophytes | *Emiliania huxleyi* |
|  | *Pavlova lutheri* |
|  | *Phaeocystis antarctica* |
|  | *Phaeocystis globosa* |
| Rhizaria | *Bigelowiella natans* |
| Excavates | *Andalucia godoyi* |
|  | *Reclinomonas americana* |
|  | *Malawimonas jakobiformis* |
|  | *Naegleria gruberi* |
| Alveolates | *Tetrahymena thermophila* |
|  | *Plasmodium falciparum* |
|  | *Karlodinium veneficum* |
|  | *Theileria parva* |
